# Highly active and thermostable submonolayer La(NiCo)O_Δ_ catalyst stabilized by a perovskite LaCrO_3_ support

**DOI:** 10.1038/s42004-022-00686-4

**Published:** 2022-06-03

**Authors:** Tingting Zhao, Jiankang Zhao, Xuyingnan Tao, Haoran Yu, Ming Li, Jie Zeng, Haiqian Wang

**Affiliations:** Hefei National Laboratory for Physical Science at the Microscale, University of Science and Technology of China, 230026 Hefei, Anhui People’s Republic of China

**Keywords:** Catalytic mechanisms, Heterogeneous catalysis, Materials for energy and catalysis

## Abstract

It is important to develop highly active and stable catalysts for high temperature reactions, such as dry reforming of methane. Here we show a La(NiCo)O_Δ_ (LNCO) submonolayer catalyst (SMLC) stabilized by the surface lattice of a perovskite LaCrO_3_ support and demonstrate a Ni-Co synergistic effect. The submonolayer/support type catalyst was prepared by in-situ hydrogen reduction of a LaNi_0.05_Co_0.05_Cr_0.9_O_3_ precursor synthesized by a sol-gel method. The LNCO-SMLC is highly active and very stable during a 100 h on stream test at 750 °C under the reaction conditions of dry reforming of methane. The catalyst also shows good anti-coking ability. We found that the synergistic effect between Ni and Co atoms in LNCO-SMLC remarkably improved the thermostability of the catalyst. This work provides a useful concept for designing atomically dispersed catalysts with high thermostability.

## Abstract (web summary)

Dry reforming of methane into syngas typically requires high temperatures, which can cause aggregation and deterioration of the catalyst. Here, the authors report a lanthanum nickel oxide catalyst prepared by in situ hydrogen reduction of LaNi_0.05_Co_0.05_Cr_0.9_O_3_ on a LaCrO_3_ perovskite support that remains stable for over 100 h at 750 °C.

## Introduction

Dry reforming of methane (DRM) is an important reaction that converts CH_4_ and CO_2_, two atmospheric greenhouse gases, into valuable syngas (H_2_ + CO)^1^. DRM is featured by high temperature (700–1000 °C) and reducing reaction atmosphere. Thermodynamically, a few side reactions accompany the main DRM reaction, such as reverse water-gas shift reaction (RWGS, CO_2_ + H_2_ = CO + H_2_O), CH_4_ decomposition reaction (CH_4_ = 2H_2_ + C), and Boudouard reaction (2CO = CO_2_ + C). Thus, besides providing high activity and selectivity, a DRM catalyst should also be sintering resistant and coking resistant. The harsh reaction conditions of DRM make it a representative probe reaction to evaluate the effectiveness and robustness of a catalyst.

Perovskite oxides with the formula of ABO_3_ are, in general, thermally stable at elevated temperatures. They are widely used as supports or precursors to fabricate metal-support catalysts^2–4^. Recently, experimental and theoretical studies show that noble-metal single atoms, such as Pt and Au, can be effectively anchored on perovskite supports such as LaFeO_3_ and SrBO_3_ (B = transition metals)^5–7^. Moreover, a perovskite itself may be catalytically active^8–10^. Transition metal atoms at the B-site are generally believed to be active owing to the d orbitals, while the A-site metals are less decisive^11^. Suntvich et al. reported that perovskite Ba_0.5_Sr_0.5_Co_0.8_Fe_0.2_O_3-δ_ (BSCF) catalyzes the OER with intrinsic activity that is at least an order of magnitude higher than that of the state-of-the-art iridium oxide catalyst in alkaline^12^. They proposed that the e_g_ orbital of surface transition metal ions participates in σ-bonding with a surface-anion adsorbate, and the e_g_ filling can greatly influence the binding of reaction intermediates to the oxide surface and thus the activity. Kim et al.^13^ demonstrated that La_0.9_Sr_0.1_CoO_3_ has higher activity than a commercial Pt-based catalyst for NO oxidation. It is also reported that perovskite La_0.75_Sr_0.25_Cr_0.5_Mn_0.5_O_3-δ_ (LSCM) can be used as an anode material for solid oxide fuel cells and shows high activity for CH_4_ oxidation^14^.

In an ideal perovskite, the larger A-site cations occupy the cubic center, while the smaller B-site cations, octahedrally coordinated to six oxygen anions each, occupy the corner sites with the BO_6_ octahedra arranged in a corner-shared configuration. Thus, a perovskite can be considered a structure constructed by BO_6_ superatoms with A-site atoms filled in between. In this sense, a perovskite is a native atomically dispersed catalyst with the catalytically functional B-site atoms embedded in BO_6_ octahedra and separated by A-site cations. The chemical (e.g., activity and selectivity) and physical (e.g., stability) properties of perovskites can be tuned conveniently by substitutional doping or creating vacancies at both the cation and anion sites.

It is proved that monovalent or low-valent Ni^δ+^ and Co^δ+^ are catalytically active owing to the partially occupied 3d orbitals^15–18^. To create catalytically active open sites with exposed Ni 3d orbitals accessible for reactants, we may simply intend to tune the NiO_6_ superatoms to NiO_x_ (x < 6) by introducing oxygen vacancies into the Ni-containing perovskite, e.g., LaNiO_3_. However, reducible perovskites containing catalytic active metal atoms, such as LaNiO_3_ or LaCoO_3_, will be over-reduced to metallic Ni or Co nanoparticles supported on La_2_O_3_ (or its derivates) under the harsh DRM reaction conditions. On the other hand, irreducible perovskites, such as LaCrO_3_, are catalytically inert. Is it possible to stabilize the catalytically active B-site Ni or Co atoms in their low-valent cationic states by preventing the perovskites from over-reducing? The fact that a perovskite can accommodate a large number of oxygen vacancies (V_O_), especially on the surface, provides the primary possibility. Thus, stabilizing the catalytically active reducible perovskite (LaNiO_Δ_ and/or LaCoO_Δ_) by stable irreducible perovskite support (such as LaCrO_3_) is interesting and attractive. In recent work, we demonstrated that bimetallic Ni–Co catalysts supported on La_2_O_3_–LaFeO_3_ exhibit higher activity and coking resistance than its monometallic (Ni or Co) counterparts for DRM reactions, which should be essentially attributed to the synergistic effect between Ni and Co^2^. We will show that such a synergistic effect also plays an important role in stabilizing SMLCs.

In this work, we report the construction of a novel submonolayer catalyst: La(NiCo)O_Δ_ submonolayer (LNCO-SML) stabilized on the surface of a perovskite LaCrO_3_ support. We prove that LNCO-SML is highly active and stable at elevated temperatures. The formation and stabilization mechanisms of the LNCO-SML and Ni–Co synergistic effect were discussed by comparative studies between LaNi_0.05_Co_0.05_Cr_0.9_O_3_ and its single metallic counterparts, LaNi_0.1_Cr_0.9_O_3_ and LaCo_0.1_Cr_0.9_O_3_.

## Results

The LaNi_0.05_Co_0.05_Cr_0.9_O_3_, LaNi_0.1_Cr_0.9_O_3_, and LaCo_0.1_Cr_0.9_O_3_ catalysts were activated by H_2_ reduction before being used for DRM reaction. As we will prove in the following content, our samples can be seen as catalytically active SMLs supported on perovskite LaCrO_3_ grains. For the convenience of discussions, we use the corresponding SMLs to designate the samples. The LaNi_0.05_Co_0.05_Cr_0.9_O_3_, LaNi_0.1_Cr_0.9_O_3_, and LaCo_0.1_Cr_0.9_O_3_ samples are denoted as LNCO, LNO, and LCO, respectively. We use the suffixes, -F, -R, and -U, to denote the fresh, H_2_-reduced, and used samples, respectively. The number following the abbreviations of used samples describes the time on stream (TOS) in hours. Thus LNCO-U100 and LCO-U100 represent the used LNCO and LCO samples after 100 h on stream tests, and LNO-U5 and LNO-U24 represent the used LNO samples after 5 h and 24 h on stream tests, respectively.

### Catalytic performance

**Fig. 1:**
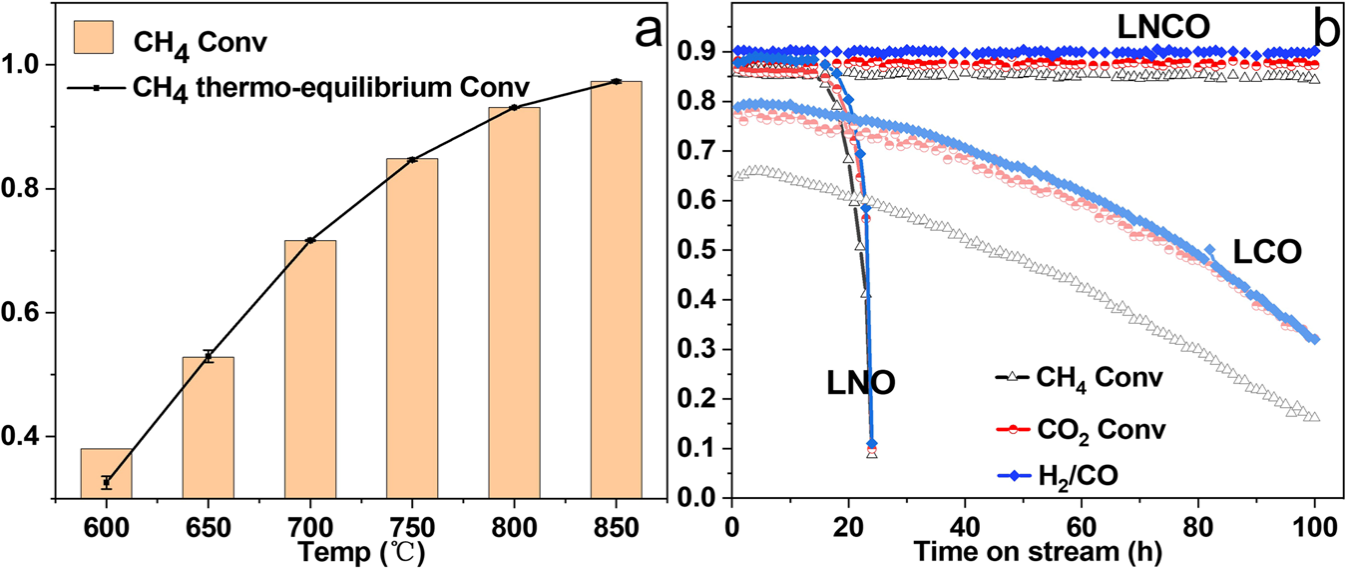
DRM performance over LNCO, LNO, and LCO catalysts. **a** Temperature-dependent CH_4_ conversion between 600 and 850 °C over LNCO catalyst, **b** DRM performance over LNCO, LNO, and LCO catalysts at 750 °C. Conditions: CH_4_:CO_2_ = 1:1, total flow rate = 60 sccm (GHSV = 1.2 × 10^4 ^mL g_cat_^−1^ h^−1^). LNCO, LNO, and LCO represent LaNi_0.05_Co_0.05_Cr_0.9_O_3_, LaNi_0.1_Cr_0.9_O_3_, and LaCo_0.1_Cr_0.9_O_3_ catalysts, respectively.

The temperature-dependent CH_4_ conversions over LNCO between 600 and 850 °C (Fig. 1a) reveal that the LNCO catalyst has high activities, which are close to the thermodynamic equilibrium values^19–22^ (without carbon deposition) under the specific experimental conditions. The long-term performance of LNCO was performed at 750 °C as shown in Fig. 1b. The CH_4_, CO_2_ conversions, and H_2_/CO ratio over LNCO are 85%, 88%, and 0.90, respectively. These results indicate that the LNCO catalyst effectively promoted the main DRM reaction. The H_2_/CO ratio is smaller than 1 is due to the RWGS reaction. The effective catalysis of LNCO on the main DRM reaction is also reflected by the high selectivity of H_2_ and CO (Supplementary Fig. 1), which are 0.94 and 0.99, respectively. Especially, the selectivity of CO is very close to 1 suggesting that side reactions of carbon deposition (CH_4_-decomposition reaction and Boudouard reaction) hardly happened. The LNCO catalyst is also very stable, and no degradation can be detected during a 100 h on stream test.

For comparison purposes, the conversions and H_2_/CO ratio over LNO and LCO are also illustrated in Fig. 1b. The conversions of CH_4_ and CO_2_ over LNO catalyst are stable at around 85 and 86% in the initial 15 h but quickly decrease to below 10% at 24 h. On the other hand, the conversions of CH_4_ and CO_2_ over LCO decrease slowly and continuously from 67 and 78% to 16 and 32%, respectively, during a 100 h on stream test. The conversions of CH_4_ (Fig. 1a) and CO_2_ (Supplementary Fig. 1a) over LNCO are in thermoequilibrium. Meanwhile, the H_2_/CO ratio (0.9) of LNCO is also very close to the thermoequilibrium value (0.92)^21^. It is seen from Fig. 1b that the decrease in the H_2_/CO ratio of LNO and LCO happened simultaneously with the decrease in the CH_4_ and CO_2_ conversions. The decrease of the CH_4_ and CO_2_ conversions over LNO and LCO to below the thermoequilibrium value indicates the DRM reaction switches from the thermodynamic-control process into a kinetic-controlled process. It is reported that Ni is highly active for RWGS reaction, and the RWGS is very fast. It immediately reaches thermoequilibrium in a wide temperature range (e.g., above 500 °C, depending on the contact time, etc.)^23,24^. Or in other words, the RWGS is less sensitive to the catalysts’ activity at high temperatures. In our experimental conditions (750 °C), the rapid decrease in the H_2_/CO ratio of LNO and LCO should be dominated by the degradation of the catalyst, which reduces the amount of DRM-generated H_2_ and CO. In this case, the less catalyst-dependent RWGS reaction will consume a relatively larger proportion of the total H_2_ and add a more significant amount of CO to the total. Thus, LNO and LCO show a rapid decrease in the H_2_/CO ratio during the reaction. On the other hand, the LNCO could suppress the RWGS reaction very well is because the catalyst is stable and the conversions of CH_4_ and CO_2_ and H_2_/CO ratio maintain at a high level.

The different performance of LNCO, LNO, and LCO indicates that the synergistic effect between Ni and Co in LNCO not only keeps the high activity but also makes the catalyst thermostable at high temperatures. We also synthesized NiCo@LaCrO_3_ with the same nominal composition to LNCO by preparing the LaCrO_3_ support first and then depositing Ni/Co ions by incipient wetness impregnation. However, the catalytic performance is very different from LNCO, LNO, and LCO prepared by the sol-gel combustion method. The conversions of CH_4_ and CO_2_ over NiCo@LaCrO_3_ are lower than LNCO and decrease fast in a slowdown trend (See Supplementary Fig. 2), which is in accord with the sintering degradation behavior of metal-support catalysts^25^. As a comparison, the conversions of CH_4_ and CO_2_ over LNO and LCO catalysts decrease in an accelerated way with time on stream. Such a NiCo@LaCrO_3_ metal-support catalyst is less stable than LNCO.

The high catalytic activity and very stable long-term performance differ the LNCO catalyst from usual metal-support catalysts reported in literatures^26–30^, especially if we consider the relatively low Ni loading (1.3 wt%) and low specific surface area (10.0 m^2^ g^−1^) of LNCO-F. As a relevant example, we reported in a recent work that the CH_4_ and CO_2_ conversions over the metal-support type catalyst, Ni–Co/La_2_O_3_-LaFeO_3_ with 7.2–10.8 wt% Ni loading and ~10 m^2^ g^−1^ specific surface area, are 70 and 80%, respectively, at 750 °C under the same DRM conditions^2^, which is much lower than those over LNCO. Moreover, the degradation behavior of LNO and LCO cannot be explained by metal-support catalysts. The conversions of CH_4_ and CO_2_ over LNO and LCO catalysts decrease in an accelerated way with time on stream. In contrast to the LNO and LCO, the decrease in conversions of CH_4_ and CO_2_ over NiCo@LaCrO_3_ shows a slowdown trend (See Supplementary Fig. 2), which is in accord with the sintering degradation behavior of metal-support catalysts^25^. The high catalytic activity of LNCO implies that the number of active sites or the dispersion of Ni and Co on the surface of the catalyst is high. The long-term stable performance at 750 °C suggests that the LNCO catalyst is thermostable, thus catalyst sintering and carbon deposition should be small.

### Crystalline structure and reducibility

**Fig. 2:**
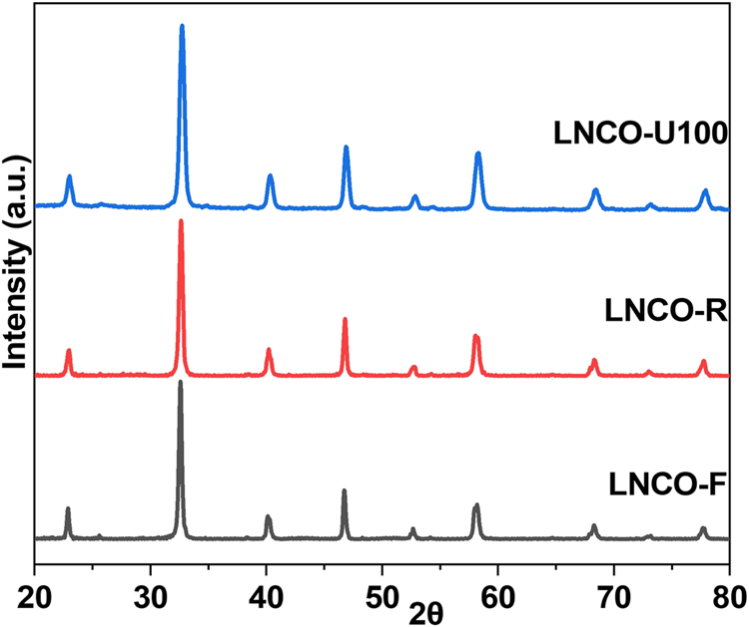
XRD patterns of perovskite LNCO samples. The XRD patterns are normalized. LNCO-F, LNCO-R, and LNCO-U100 represent fresh, H_2_-reduced, and used after 100 h time on stream LaNi_0.05_Co_0.05_Cr_0.9_O_3_ samples, respectively.

The XRD pattern of LNCO-F is dominated by a well-defined perovskite phase with space group Pbnm (LaCrO_3_: JCPDS 71-1231) (Fig. 2). No impurities, such as NiO, Co_2_O_3_, Co_3_O_4_, etc. can be detected, indicating that nickel and cobalt atoms are well incorporated into the crystalline perovskite lattice. After LNCO-F undergoes H_2_ reduction and DRM test, the diffraction patterns and peak positions of LNCO-R and LNCO-U100 samples are almost the same as those of LNCO-F. No separated Ni, Co, or NiCo alloy phases can be detected in LNCO-R and LNCO-U100 should be because the amount of the metallic phases is very small and below the detection limit of XRD. The XRD patterns of LNO and LCO samples (Supplementary Fig. 3) are similar to those of LNCO, in which all the patterns are dominated by the perovskite phase with space group Pbnm (LaCrO_3_: JCPDS 71-1231). The XRD single-phase nature of the used sample indicates that the perovskite crystalline structure of LNCO, LNO, and LCO is very stable, and it is difficult to exsolve the Ni and Co atoms from the perovskite matrix under DRM conditions.

Supplementary Fig. 4 shows the TG and DTG profiles of LNCO-F, LNO-F, and LCO-F measured by TPR. The overall mass losses of all the samples are less than 1.0% as the temperature increases from 30 to 1200 °C, indicating that the samples are very stable under hydrogen reduction. Nevertheless, a reduction step at about 300–400 °C is resolved in each of the mass-loss profiles, which can be assigned to the reduction of Ni^3+^ to Ni^2+^ and/or Co^3+^ to Co^2+^ ^2,31–34^. The mass losses at 300–400 °C are about 0.06% for LNCO-F, 0.26% for LNO-F, and 0.06% for LCO-F, which are smaller than the theoretical value of 0.33% for a full reduction to Ni^2+^ and Co^2+^, indicating that not all the Ni^3+^ and Co^3+^ cations are reduced to Ni^2+^ and Co^2+^ in this reduction step. The reduction step at about 500 °C should be assigned to the reduction of Cr^6+^ to Cr^3+^ ^35^ because of the existence of a small amount of Cr^6+^ cations in the fresh samples, as evidenced by XPS (Supplementary Fig. 6). It is interesting to note that no relevant reduction step of Ni^2+^ to Ni^0^ and/or Co^2+^ to Co^0^ can be observed, suggesting that the Ni^2+^ and Co^2+^ ions in LNCO, LNO, and LCO are stable and hard to be reduced to Ni^0^ and Co^0^. As the temperature increases to above 500 °C, LNO-F further reduces slowly and continuously. This should be related to the slow release of Ni^3+^ from the inside of the perovskite grains and, probably, further reduction of Ni^2+^ to its lower valence state Ni^δ+^ or Ni^0^, especially near the surface regions. Similar TPR profiles were also reported by Stojanović et al., and the authors concluded that LaNi_x_Cr_1-x_O_3_ compounds with x < 0.5 do not reduce to nickel metal in the H_2_ atmosphere at <900 °C^36^.

### Surface Ni distribution and electronic interactions

**Fig. 3:**
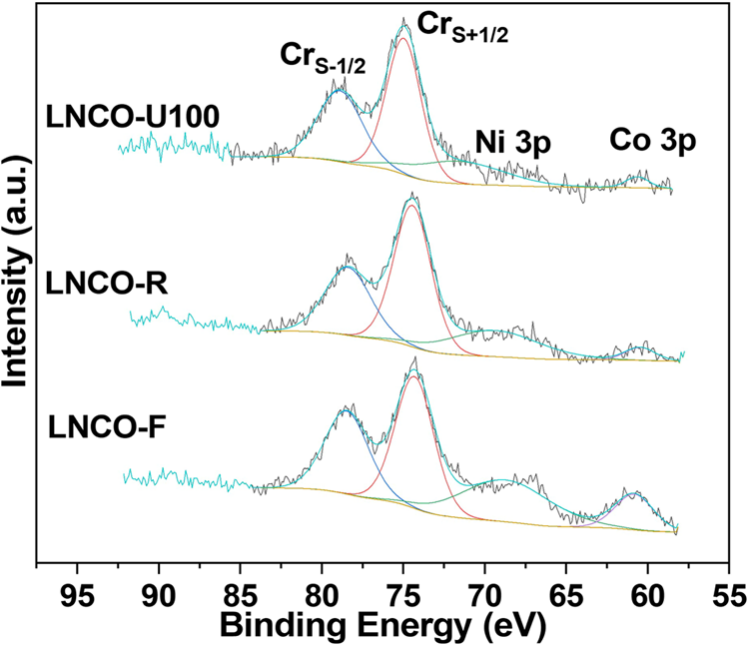
Ni 3p, Co 3p, and Cr 3s XPS spectra of LNCO samples. Cr_S+1/2_ and Cr_S-1/2_ denote intra-atomic multiplet splitting of Cr 3s with the remaining 3s electron coupled parallel and antiparallel to the 3d electrons, respectively. LNCO-F, LNCO-R, and LNCO-U100 represent fresh, H_2_-reduced, and used after 100 h time on stream LaNi_0.05_Co_0.05_Cr_0.9_O_3_ samples, respectively.

The surface distribution of Ni and Co is essential since it reflects the number of active sites. We use the Cr 3s XPS spectrum to determine the surface content of Ni and Co because the most intensive Ni 2p_3/2_ peak overlaps heavily with La 3d_3/2_. Figure 3 shows that the Cr 3s spectrum of LNCO has two well-separated peaks at about 74.4 eV (denoted as Cr_S+1/2_) and 78.6 eV (denoted as Cr_S-1/2_) due to intra-atomic multiplet splitting^37^. They are close to the Ni 3p peak (BE ≈69.5 eV) and Co 3p peak (BE ≈60.5 eV) and thus can be used as an internal reference to determine the Ni and Co contents. Both Ni and Co contents show a decreasing trend with the sample sequence of LNCO-F → LNCO-R ≈ LNCO-U100 (see Fig. 3 and Supplementary Table 1). The Ni and Co contents detected by XPS depend on the probing depth (2–5 nm for solids^38^) and the distribution form of Ni and Co. In LNCO-F, Ni and Co atoms are distributed homogeneously within the perovskite lattice (see Supplementary Fig. 7), and thus XPS will “see” all the Ni and Co atoms within the probing depth. Once the Ni and Co atoms in the surface layer aggregate into 3D nanoparticles with diameters larger than the probing depth, the Ni and Co contents detected by XPS will reduce. In the case of the coverage of Ni and Co nanoparticles are small in terms of projected area on the perovskite surface (fewer and larger NiCo 3D nanoparticles, see Supplementary Figs. 8 and 9), the surface Ni and Co contents detected by XPS mainly depend on the atomically dispersed Ni and Co atoms on the perovskite surface. Thus, the XPS results indicate that the number of atomically dispersed Ni and Co atoms on the surface of LNCO-R is smaller than that of LNCO-F but close to or slightly larger than that of LNCO-U100. The decrease in the Ni and Co contents also implies a transverse migration of the atomically dispersed Ni and Co atoms when they aggregate into 3D nanoparticles.

The Cr 3s XPS spectra also reflect the interaction between the B-site Ni, Co, and Cr atoms. The multiplet splitting of Cr 3s arises from the exchange interaction between the spin of the remaining Cr 3s electron and local unpaired valence 3d electrons, and the magnitude of the energy separation ΔE = (2 S + 1) G^2^(3s, 3d), where S is the total spin of the local valence 3d electrons, and G^2^(3s, 3d) the exchange integral between the 3s and 3d orbitals^38–42^. For the specific valence state of Cr^3+^, ΔE also depends on the electronegativity of the ligand because the covalency between Cr^3+^ and ligand (O^2−^ in the present case) affects the overlap (or the exchange integral) between the 3s and 3d orbitals^41^.

Supplementary Table 1 shows that ΔE reduces from 4.2 eV for LNCO-F to 4.0 and 3.9 eV for LNCO-R and LNCO-U100, respectively. The similar changing trend of ΔE and the Ni and Co contents (see Fig. 3 and Supplementary Table 1) with the sample sequence (LNCO-F, LNCO-R, and LNCO-U100) suggests that there are some kinds of interactions between the B-site Cr and Ni or Co atoms. For Cr compounds (LaNi_0.05_Co_0.05_Cr_0.9_O_3_ in the present case), as a first-order approximation, ΔE depends on the total 3d ground-state spin^38,40,43^. The observed ΔE (around 4 eV) for the LNCO samples agrees well with the reported values for Cr(III) compounds^40,41,43^, confirming that the Cr ions are in the Cr^3+^ oxidation state. The very small amount of Cr^6+^ in LNCO-F (see Supplementary Fig. 6) does not contribute to the exchange splitting because S = 0 for the 3d^0^ electron configuration. The larger ΔE observed in LNCO-F should be induced by the ligand effect^41^. Charge transfer from Ni^3+^ (3d) or Co^3+^ (3d) through O^2-^ (2p) to Cr^3+^ (3d) will push the Cr 3d orbital inward (more localized), thus increasing the overlap (the exchange integral) between the Cr 3s and 3d orbitals. In this sense, Ni or Co can be considered as an electron donor of LaCrO_3_. According to Saitoh’s work^44^, the doping electrons in LaCrO_3_ have more Cr 3d character and less O 2p character, which means that the doped electrons from Ni or Co mainly go to the Cr 3d orbitals. Thus, the decreases of ΔE in the LNCO-R and LNCO-U100 samples can be explained by the decrease in the atomically dispersed Ni and Co contents because fewer Ni^3+^ and Co^3+^ ions are available for the charge transfer. Nevertheless, the very close ΔE’s between the LNCO-R and LNCO-U100 samples indicate that the atomically dispersed Ni and Co atoms are very stable over the 100 h on stream test.

Similar XPS features were also observed in LNO and LCO (see Supplementary Fig. 5 and Supplementary Table 1). Therefore, the above discussions can also be applied to LNO and LCO.

### Structure and composition of SMLs

**Fig. 4:**
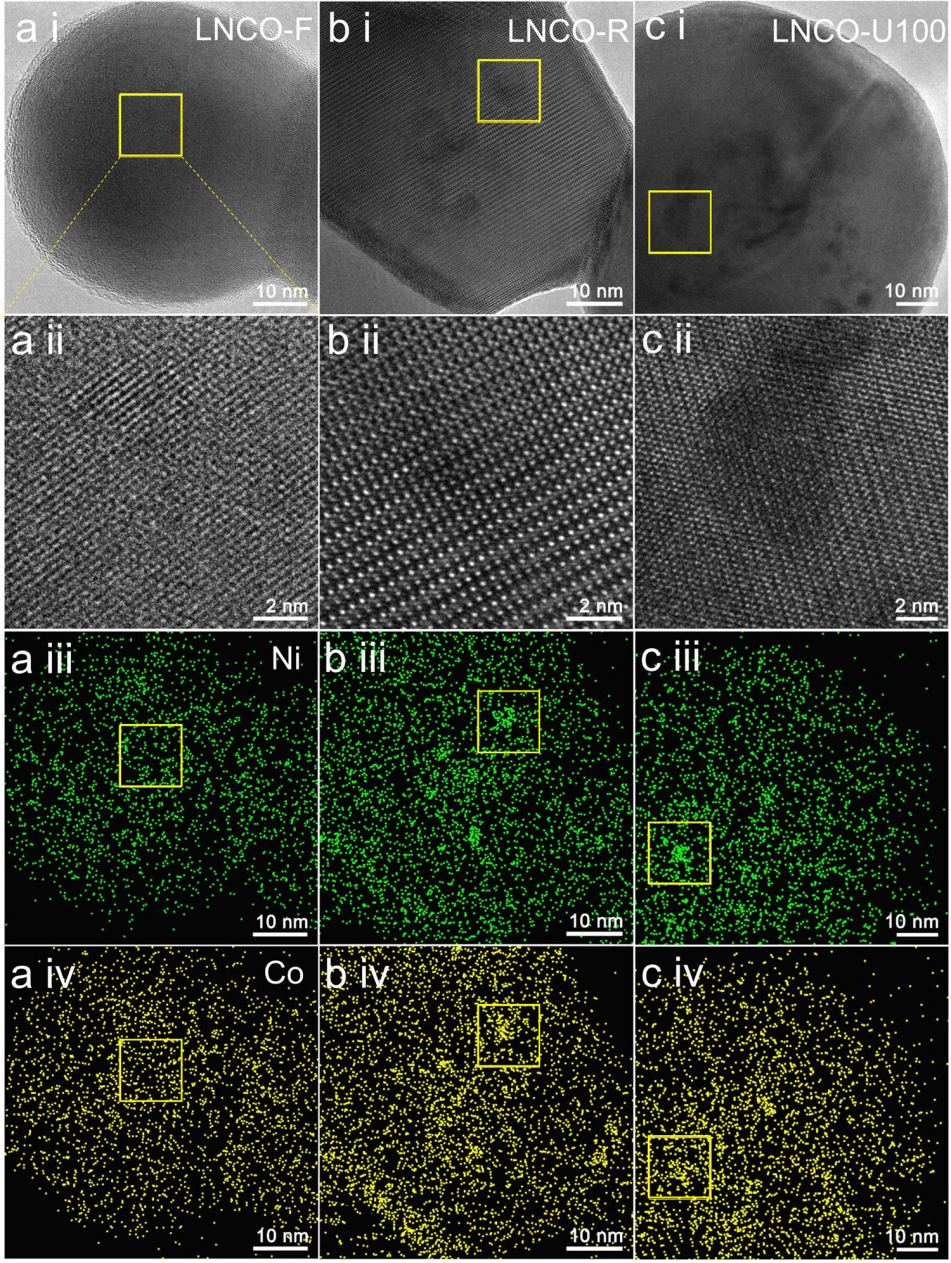
HR-TEM images and EDS Mapping of Ni and Co of LNCO samples. **a** LNCO-F, **b** LNCO-R, and **c** LNCO-U100 represent fresh, H_2_-reduced, and used after 100 h time on stream LaNi_0.05_Co_0.05_Cr_0.9_O_3_ samples, respectively. The 1st row: HR-TEM images, the 2nd row: magnified HR-TEM images corresponding to the yellow squares in the 1st row, the 3rd row: EDS Mapping of Ni corresponding to the HR-TEM images in the 1st row, and the 4th row: EDS Mapping of Co corresponding to the HR-TEM images in the 1st row. The regions marked by yellow squares in (**b**, **c**) are Ni-rich and Co-rich.

Typical HR-TEM images of the LNCO samples (Fig. 4) reveal that the catalyst is composed of well-crystallized grains, and the spacing between the fringes of the grains can be indexed to the perovskite phase as determined by XRD. EDS mapping of LNCO-F shows homogeneous distributions of all the La, Cr, Ni, Co, and O elements, indicating that Ni and Co atoms dissolve uniformly in the LaCrO_3_ perovskite lattice (Fig. 4 and Supplementary Fig. 7). LNCO-R and LNCO-U100 differ from LNCO-F in that we can observe many dark regions (marked by yellow square) in the HR-TEM images (Fig. 4bi and 4ci) and the dark regions are Ni- and Co-rich (denoted as NiCo-rich regions) (Fig. 4biii, biv, ciii, and civ). It is interesting to note that these NiCo-rich regions share the same lattice structure with the LaCrO_3_ grains, indicating that the Ni and Co atoms occupy the perovskite lattice sites, but do not exist in the form of metallic Ni–Co alloy nanoparticles. On the other hand, no aggregation of La occurs in LNCO-R and LNCO-U100 (see Supplementary Figs. 8 and 9), suggesting that La distributes uniformly in these samples but did not segregate into the La_2_O_3_ phase or its derivatives. This further confirms that the NiCo-rich regions are, essentially, perovskite-type LaNiCoO_Δ_ (LNCO). No protrusions can be found at the projected boundaries of the grains in the LNCO-R and LNCO-U100 samples (Fig. 4) suggesting that the LNCO is in the two-dimensional (2D) form. This resulted from the reducible nature of LNCO. As we know, 3D bulk perovskite LaNiO_3_ and LaCoO_3_ are not stable and will decompose completely into metallic Ni and Co nanoparticles and La_2_O_3_ under H_2_-reduction and DRM conditions^2^. The stable existence of submonolayer LNCO should be primarily attributed to the strong interaction between the LNCO and the LaCrO_3_ support. Moreover, the overlap of the Ni-rich and Co-rich regions in Fig. 4 indicates that the interactions between the B-site Ni and Co atoms make LNCO more stable than individual LaNiO_Δ_ or LaCoO_Δ_. Thus, we know that the synergistic effect between Ni and Co further stabilizes the LNCO. We believe that the LNCO submonolayer is V_O_-rich based on the understanding that there are plenty of oxygen vacancies on the surface of reducible perovskites. Such LNCO-SML stabilizes the catalytically active Ni and Co atoms in their low-valent states (Ni^δ+^ and Co^δ+^). Thus, the LNCO-SMLs should be responsible for the high catalytic activity of the LaNi_0.05_Co_0.05_Cr_0.9_O_3_ catalyst. We can also observe some metallic NiCo nanoparticles with lateral sizes of more than 50 nm in the LNCO-U100 sample (see Supplementary Fig. 9b). However, the number of NiCo nanoparticles per unit area on the surface is very low as compared with that in the traditional metal-support catalysts^2^. The few but large NiCo nanoparticles cannot explain the very high catalytic activity of the LNCO-U100 sample. Thus, we conclude that the high catalytic activity of LNCO-U100 comes from the LNCO-SMLs but not from the metallic NiCo nanoparticles.

**Fig. 5:**
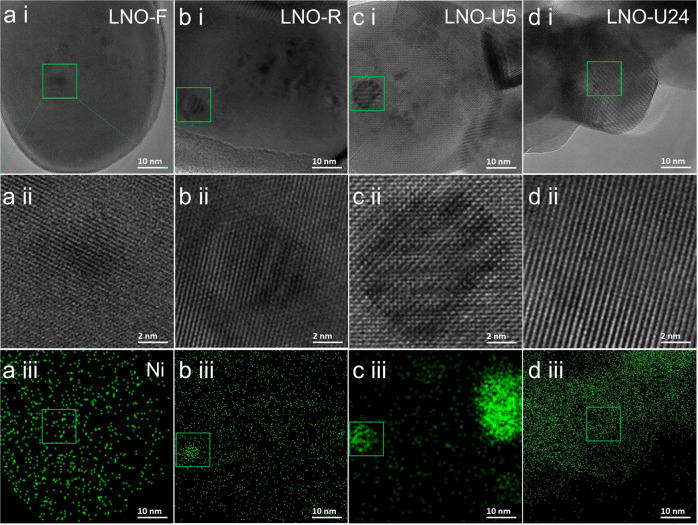
HR-TEM images and EDS Mapping of Ni of LNO samples. **a** LNO-F, **b** LNO-R, **c** LNO-U5, and **d** LNO-U24 represent fresh, H_2_-reduced, used after 5 h time on stream and used after 24 h time on stream LaNi_0.1_Cr_0.9_O_3_ samples, respectively. The 1st row: HR-TEM images, the 2nd row: magnified HR-TEM images corresponding to the green squares in the 1st row, and the 3rd row: EDS Mapping of Ni corresponding to the HR-TEM images in the 1st row. The regions marked by green squares in (**b**, **c**) are Ni-rich.

To further confirm that catalysts are of SMLs/support type and the SMLs are responsible for the high activity, we also performed similar HR-TEM and EDS-Mapping analyses on the LNO samples (see Fig. 5 and Supplementary Figs. 10–13). As expected, the same SML features, including the dark regions in HR-TEM, the Ni-rich regions in the EDS-Mapping images, and the sharing of the lattice with the LaCrO_3_ support, are represented in the LNO-R and LNO-U5 samples. Thus, we can assign the SMLs to LaNiO_Δ_ submonolayers (LNO-SMLs). Such an LNO-SMLs stabilizes the catalytically active Ni atoms in their low-valent states Ni^δ+^. Thus, the LNO-SMLs should be responsible for the initial catalytic activity of the LNO catalyst. Few LNO-SMLs and more Ni nanoparticles (see Fig. 5 and Supplementary Fig. 13) in the deactivated LNO-U24 sample further prove that the high catalytic activity of LNO-U5 (see Fig. 5 and Supplementary Fig. 12) comes from the LNO-SMLs but not from the metallic Ni nanoparticles. Similar features can also be found in the LCO-U100 sample (Supplementary Fig. 14). In contrast, Supplementary Fig. 15 shows that the used NiCo@LaCrO_3_ sample is dominated by metallic Ni nanoparticles.

**Fig. 6:**
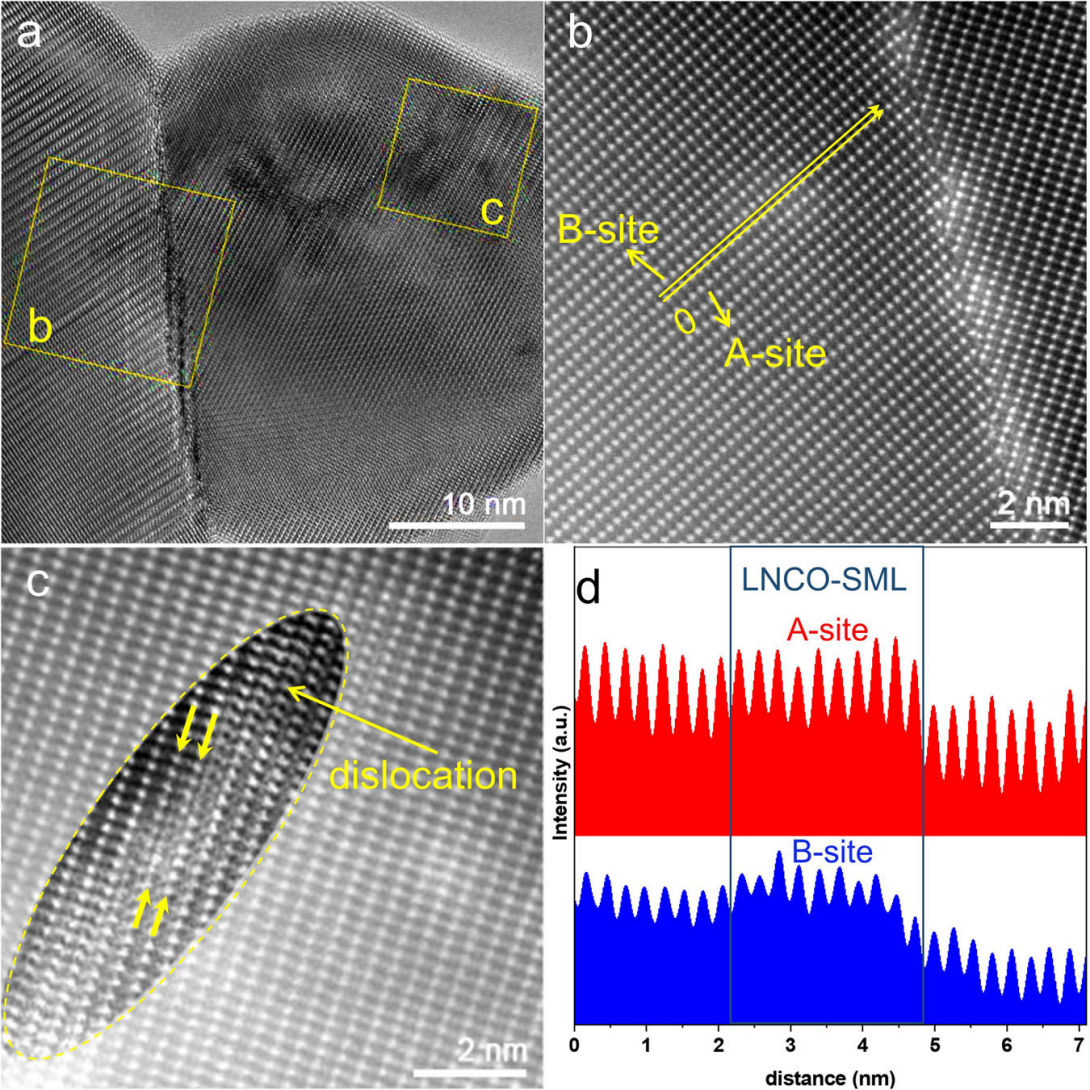
HR-TEM and HAADF images of LNCO-R sample. **a** HR-TEM, **b**, **c** HAADF images of LNCO-R sample corresponding to the yellow squares in **a**. **d** The line profile across A- and B-site elements as indicated in **b**. The contrast in the ellipse in **c** was enhanced to highlight the dislocation in LNCO-SML. The surface direction is [020]. LNCO-R represents H_2_-reduced LaNi_0.05_Co_0.05_Cr_0.9_O_3_ sample. LNCO-SML represents La(NiCo)O_Δ_ submonolayer.

To understand mechanisms for the formation of the SMLs on the LaCrO_3_ surface, we examined the HR-TEM (Fig. 6a) and Z-contrast HAADF images of LNCO-R (Fig. 6b, c) in the same selected area. It is interesting to see from Fig. 6b that there are some dark regions at the margins of SMLs (bright regions), which should be resulted from missing La, Ni, and Co atoms (NiCo-deficient regions). This phenomenon implies that the SMLs are formed by short-distance transverse diffusion of Ni, Co, and La atoms on the LaCrO_3_ surface. The line profiles across the SMLs (Fig. 6d) reveal that both A- and B-site elements show higher intensity in the LNCO-SML regions, further confirming that SMLs are La(NiCo)O_Δ_. The pronounced difference in the intensities between the SML and the NiCo-deficient regions in the line profile (Fig. 6d) should result from both the geometric and atomic factors: (1) the SMLs are at least one atomic step higher than the NiCo-deficient regions due to the out-migration of Ni/Co and La atoms, and (2) the atomic numbers of Ni and Co are higher than Cr, thus Ni and Co appear a little brighter in the HAADF images. Similar phenomena can also be observed in the LNO-R sample (Supplementary Fig. 16), confirming that the SMLs in LNO-R are LaNiO_Δ_ submonolayers.

It is also interesting to note that the SMLs tend to reside at the defect sites, such as grain boundaries (Fig. 6b) and dislocations (Fig. 6c) in the LNCO-R sample. Such kinds of solid defects interrupt the periodicity of the surface lattice, and thus, block the surface migration of Ni and Co single atoms. The presence of these defects not only enhances the anchoring of SMLs on the LaCrO_3_ surface but could also alter the electronic structures of the active Ni and Co single atoms in the catalyst. The same features of SML reserves in LNCO-U100 (Supplementary Fig. 17) indicate that the SMLs are stable during the 100 h long-term DRM test. In contrast, the metallic NiCo nanoparticles grow much larger after 100 h on stream test due to Ostwald ripening (see Supplementary Fig. 9b). On the other hand, no dislocations nor aggregation of SMLs at the grain boundaries can be observed in the less stable LNO-R sample (see Supplementary Fig. 16), proving that the Ni–Co synergistic effect plays an important role in stabilizing the SMLs.

## Discussion

### The proposed model for SMLC: activity and stability

**Fig. 7:**
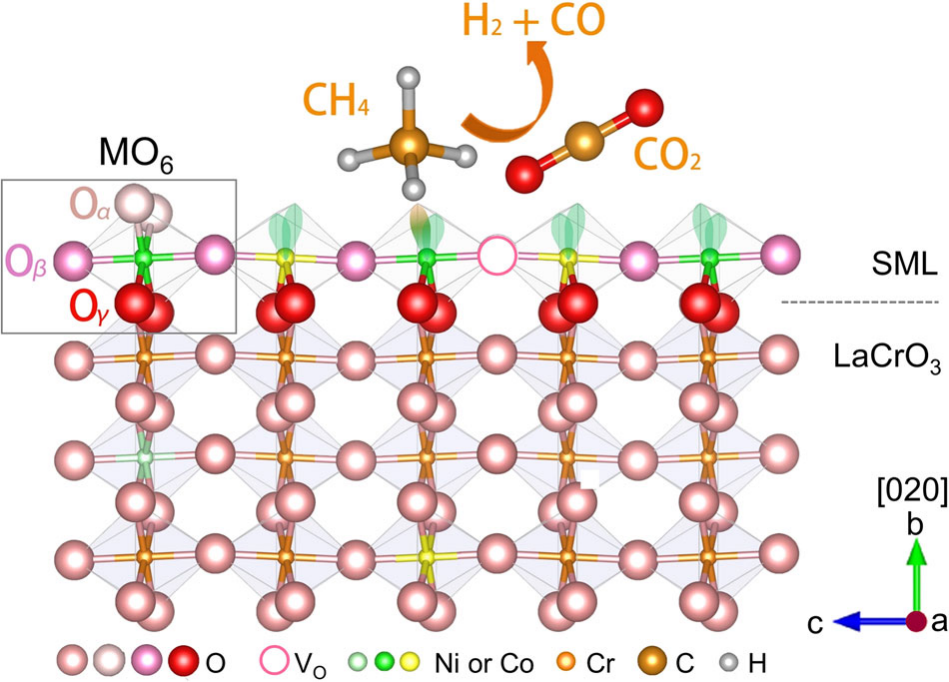
Schematic diagram of LNCO-SML stabilized by perovskite LaCrO_3_ surface lattice (space group Pbnm, La not shown). LNCO-SML represents La(NiCo)O_Δ_ submonolayer. M represents Ni or Co. The MO_6_ octahedron in the gray rectangle represents a Ni^3+^ or Co^3+^ cation coordinated to six O^2-^ anions. O_α_, O_β_, and O_γ_ have different bonding configurations with M and Cr. CH_4_ and CO_2_ are the reactants, and H_2_ and CO are the products of DRM. Losses of O_α_ and O_β_ will reduce Ni^3+^ or Co^3+^ cations to the low-valent states (Ni^δ+^ or Co^δ+^) and expose the Ni or Co 3d orbitals to the reactants (or reaction intermediates).

Based on the above experimental observations and analyses, we propose a simplified model to qualitatively illustrate the formation and catalytic activity of SMLs on the stable low index [020] surface (viewing from the [100] direction) of a perovskite LaCrO_3_ as schematically shown in Fig. 7. We start from an ideal picture that a monolayer LaMO_3_ (M = Ni or Co) is epitaxially grown on the surface of LaCrO_3_ (see the MO_6_ octahedron marked by a gray rectangle in Fig. 7). Apparently, O atoms at the six corner sites of the MO_6_ octahedron are not equivalent. The two O atoms located at the top of the octahedron (labeled as O_α_) have the lowest coordination number to M (O_α_–M) and should be the least stable ones. The two O_β_ atoms at the middle of the octahedron coordinate to two M atoms each (M–O_β_–M), should be more stable than O_α_ due to the increased coordination number. O_γ_, located at the bottom of the octahedron, directly connects to the surface of LaCrO_3_ with the bonding configuration of M–O_γ_–Cr. O_γ_ should be the most stable ones owing to the strong bonding with less electronegative Cr^3+^.

Upon H_2_ reduction, O_α_ ions are removed from the lattice, leaving two electrons behind, and simultaneously, M^3+^ ions are reduced to lower valence states (M^2+^ or M^δ+^). Once this happens, M 3d orbitals are open to the reactants, and the catalyst becomes activated (Fig. 7). In the process of catalyzing a DRM reaction, it is very likely that the metastable O_β_ is active and involved in the elementary reactions. It is widely accepted that many perovskites are characterized by active lattice oxygen that may take part in the elementary reactions of DRM^31,45^. This means that O_β_ should be exchangeable with the oxygen species deduced from CO_2_. Such kind of active oxygen helps activate CO_2_ and suppress carbon deposition. Certainly, there are possibilities that O_β_ escapes from the lattice site and introduces additional oxygen vacancies (V_O_ in Fig. 7), and thus, further reduces M^2+^ to M^δ+^ (0 < δ < 2). Many reported works correlated the active catalytic sites to Ni^δ+^ for Ni-based catalysts^15,16,46^. Yang et al.^15^ proved that monovalent Ni(I) atoms anchored on the N-doped graphene matrix are catalytically active owing to the partially occupied 3d orbitals. However, Ni(I) ions are frequently thermally unstable^15,46^. In the present work, the stable O_γ_ in SML should be responsible for the stabilization of the Ni(I) (or Ni^δ+^). In our case, we proposed that the high activity of LNCO comes from the atomically dispersed low-valent Ni^δ+^ (and Co^δ+^) metal cations with open 3d orbitals and filled e_g_ electrons. Considering that Ni is more active than Co for DRM, we think the catalytic activity of LNCO is dominated by Ni, while Co mainly contributes to enhancing the stability of the submonolayer structure. Moreover, the A-site La^3+^ and charger transfer between Ni, Co, and Cr should also facilitate the anchoring of SML on LaCrO_3_ support^47^. It is reported that Pt atoms were firmly anchored on the surface of SiC nanocrystallites by forming a 2D superstructure^48^. In analogy, Ni and Co atoms in the 2D SML superstructure should be more stable than separated single Ni or Co atoms on the surface. The increased mixing entropy of Ni–Co in the submonolayer should be also helpful for the stabilization of LNCO-SML.

### Synergistic effect between Ni and Co

The LNCO catalyst shows higher thermostability as compared to LNO and LCO (see Fig. 1b). It is known that LaCoO_3_ is more stable than LaNiO_3_ in reducing atmosphere^49^ and the affinity of Co to oxygen is stronger than Ni^2^. In the 2D case, the stronger affinity of Co to oxygen should help stabilize O_β_ in the Co–O_β_–Ni bonding configuration (see Fig. 7) and prevent the LNCO-SML from breaking up. Similarly, a stronger Co–O_γ_ bond should facilitate the anchoring of LNCO-SML to the surface lattice of LaCrO_3_. The coexistence of Co and Ni in LNCO-R also favors the formation of solid defects (point defects and dislocations etc.) owing to the different ionic radii in reducing atmosphere. It is known that solid defects could facilitate the anchoring of single-atom sites by tuning their surrounding electronic structure and coordination environment^50,51^. Thus, the solid defects as we have seen in Fig. 6 (grain boundaries and dislocations) further enhance the stability of the LNCO-SML.

To understand the superior performance of DRM on Ni–Co/LaCrO_3_ catalyst, we performed density functional theory (DFT + U) calculations for the stability of Ni and Co atoms on our model catalysts for a few typical configurations. The calculation results show that the configuration with B-site neighboring Ni and Co atoms on the top layer (010 surface) is 0.53 eV more stable than the configuration with one Ni atom on the top surface and one Co atom in the bulk (Supplementary Fig. 18). The configuration with B-site neighboring Co, Ni, Co atoms on the top layer (001 surface) is 0.14 eV more stable than that with two Co atoms on the top surface and one Ni atom in the bulk (Supplementary Fig. 19). These results indicate that the configuration with both Ni and Co on the surface is more stable than those with only Ni or only Co atom on the surface, and the formation of the Ni–O-Co chain on the surface enhances the stability of La(NiCo)O_Δ_-SML supported on LaCrO_3_, namely the LNCO sample.

### Anti-coking property

As we have seen in Fig. 1, the catalytic performance of LNCO is stable over the long-term 100 h on stream test, and it is hard to find any filamentous carbon in the TEM images (See Supplementary Fig. 9). These results indicate that the LNCO-SMLs have a good anti-coking property. Kim et al.^52^ studied the effect of Ni particle size on coking for DRM reaction and found that Ni particles smaller than 7 nm can effectively suppress the formation of filamentous carbon and highly dispersed metal particles showed a remarkably low coking rate. It is reported that the absence of adjacent active metal atoms (Ni or Co) could prevent the C–C coupling or coking. Such a mechanism was used to explain the coking resistance of Fe single atoms embedded in silica matrix for the high-temperature direct conversion of methane to ethylene^53^. It is also reported that Ni single-atom catalysts favor partial CH_4_ dehydrogenation and thus the complete decomposition to C is suppressed^1^. The higher oxygen affinity of Co also favors enhancing the coking resistance^2^.

Nevertheless, we did detect a small amount of deposited carbon in the LNCO-U100, LNO-U24, and LCO-U100 samples. Our TPO analysis shows the average carbon deposition rates of LNCO-U100, LNO-U24, and LCO-U100 are 0.87, 0.25, and 0.09 mg_C_ g_cat_^−1^ h^−1^, respectively (Supplementary Fig. 20). Considering the coexistence of SMLs and a few large Ni(Co) nanoparticles (>50 nm), the deposited carbon in the SML/LaCrO_3_ catalysts should be correlated to the metallic Ni(Co) nanoparticles. Thus, we tend to conclude that the SMLs not only have high catalytic activity and thermostability but also have a good anti-coking property.

### Conclusions

We successfully constructed a novel SMLC by in situ hydrogen reduction of a perovskite LaNi_0.05_Co_0.05_Cr_0.9_O_3_ precursor synthesized by the sol-gel method. The LNCO-SML is highly active and very stable over 100 h on stream test at 750 °C under DRM conditions. We demonstrated that the atomically dispersed Ni and Co atoms in LNCO-SMLC stabilized by irreducible perovskite LaCrO_3_ hold the high activity of LNO with remarkably improved thermostability. The synergistic effect between Ni and Co further enhances the anchoring of LNCO-SMLs on the surface lattice of a perovskite LaCrO_3_ support by introducing stronger Co–O bonds and more solid defects.

This work provides a useful concept for designing atomically dispersed catalysts with high thermostability. There is plenty of room to explore SMLCs with a large variety of catalytic active noble or non-noble-metal atoms for other reactions.

## Methods

### Catalyst preparation

LaNi_0.05_Co_0.05_Cr_0.9_O_3_ (LNCO), LaNi_0.1_Cr_0.9_O_3_ (LN), and LaCo_0.1_Cr_0.9_O_3_ (LC) catalyst precursors were synthesized using a sol-gel self-combustion method^2,54^. All the chemicals of analytical grade were purchased from Sinopharm Chemical Agent Company. Lanthanum oxide (La_2_O_3_) was entirely dissolved in nitric acid aqueous solution. The stoichiometric ratio of nickel nitrate hexahydrate (Ni(NO_3_)_2_•6H_2_O) and/or cobalt nitrate hexahydrate (Co(NO_3_)_2_•6H_2_O), and chromium nitrate nonahydrate (Cr(NO_3_)_3_•9H_2_O) were added into the solution under constant stirring. Then, a proper amount of citric acid monohydrate (C_6_H_8_O_7_•H_2_O) was added as the complexing agent. After all the agents were entirely dissolved, ammonia solution (25% NH_3_ by weight in water) was added to adjust the pH value of the solution to 7–9. After constant stirring at room temperature for a proper time, the mixed solution was heated on a heating platform until ignited. Then the product powder was collected and calcined at 700 °C in the air for 4 h to remove residual organic chemicals. The obtained catalyst precursor was in the gray-brown spongy powder form.

The LaCrO_3_ support was synthesized by the same method as above. And the stoichiometric ratio of nickel nitrate hexahydrate (Ni(NO_3_)_2_•6H_2_O), cobalt nitrate hexahydrate (Co(NO_3_)_2_•6H_2_O) and LaCrO_3_ support were added in deionized water (1.25 wt% Ni and 1.25 wt% Co of LaCrO_3_ support). After stirring for 12 h, the mixture was dried at 80 °C for 12 h and then calcined at 700 °C in air for 4 h. The impregnated NiCo@LaCrO_3_ was obtained.

### Characterization

The crystalline phase structure of the catalyst samples was examined by an X-ray diffractometer (XRD, MXPAHF, MacScience) using Cu Kα radiation (*λ* = 1.5406 Å) over the range of 2*θ* = 20–80° at room temperature.

X-ray photoelectron spectroscopy (XPS) analysis was performed using an electron spectrometer (ESCALAB 250, Thermo-VG Scientific, USA) with an exciting source of Al Kα = 1486.6 eV.

The microstructures of samples were observed by high-resolution transmission electron microscopy (HR-TEM, Talos F200X, FEI, USA) and high-angle annular dark-field scanning transmission electron microscopy (HAADF-STEM, JEM-ARM200F, JEOL, Japan) operating at an accelerating voltage of 200 kV. The element distribution was measured by energy-dispersive X-ray spectroscopy mapping analysis (EDS**-**Mapping, Talos F200X, FEI, USA).

Temperature programmed reduction (TPR) was carried out with a simultaneous thermal analyzer (STA449F3, NETZSCH, Germany). In all, 10–15 mg powder sample was placed in an alumina crucible and degassed at 230 °C in vacuum for 1 h to remove adsorbates. After cooled down to room temperature, the sample was heated in situ to 1200 °C with a heating rate of 10 °C min^−1^. The flow rate of the forming gas (5 vol% H_2_/N_2_) was 60 sccm. We take the first-order derivative on the mass-loss curve as DTG.

Temperature programmed oxidation (TPO) was performed on the used catalysts to analyze the carbon deposition. The analysis was carried out with a simultaneous thermal analyzer (STA 449 F3, NETZSCH, Germany). 10–15 mg powder sample was placed in an alumina crucible. The sample was first heated to 800 °C under 10 sccm N_2_ protection with a heating rate of 10 °C min^−1^ to remove adsorbed gas molecules and to decompose any possible La_2_O_2_(CO_3_). After cooled down to room temperature, the sample was heated to 1000 °C in dry air (flow rate = 10 sccm) with a heating rate of 10 °C min^−1^. The mass loss detected in the high-temperature stage above 500 °C reflects the amount of deposited carbon.

The BET-specific surface areas were measured by nitrogen adsorption at liquid nitrogen temperature (77 K) using a surface area analyzer (NOVA 3200e, Quantachrome, USA). Before N_2_ adsorption, the samples were degassed at 300 °C for 3 h to remove any residual moisture and other volatiles.

### Catalytic activity tests

In all, 300 mg sample was placed in a fixed bed quartz reactor (i.d. = 6 mm) without dilution. The sample was activated in pure H_2_ at 700 °C for 1 h before catalytic tests. After purging by N_2_ for 30 min, the reactor was heated to the test temperature to carry out the catalyst activity test under a continuous feed of approximate equimolecular CO_2_/CH_4_ mixture with a flow rate of 60 mL min^−1^ without dilution. The same gaseous hourly space velocity (GHSV) of 1.2 × 10^4 ^mL g_cat_^−1^ h^−1^ was kept throughout the test. The steady-state tests were performed under atmospheric pressure at 750 °C. The reaction products were analyzed by online gas chromatography (GC9790, FULI), and the flow rate of the tail gas was measured by a soap film flowmeter. The temperature-dependent test was performed by reducing the temperature from 850 to 600 °C with a temperature step of 50 °C. At each step, the temperature was stabilized for 10 min and four data points were collected in the succeeding 120 min. The conversions of CH_4_ and CO_2_, H_2_/CO ratio, and selectivities of H_2_ and CO are defined as:

$${{{{{\rm{Conv}}}}}}\,{{{{{{\rm{CH}}}}}}}_{4}=\frac{{\left[{{{{{{\rm{CH}}}}}}}_{4}\right]}_{{{{{{\rm{in}}}}}}}-{\left[{{{{{{\rm{CH}}}}}}}_{4}\right]}_{{{{{{\rm{out}}}}}}}}{{\left[{{{{{{\rm{CH}}}}}}}_{4}\right]}_{{{{{{\rm{in}}}}}}}}\times 100 \%$$

$${{{{{\rm{Conv}}}}}}\,{{{{{{\rm{CO}}}}}}}_{2}=\frac{{\left[{{{{{{\rm{CO}}}}}}}_{2}\right]}_{{{{{{\rm{in}}}}}}}-{\left[{{{{{{\rm{CO}}}}}}}_{2}\right]}_{{{{{{\rm{out}}}}}}}}{{\left[{{{{{{\rm{CO}}}}}}}_{2}\right]}_{{{{{{\rm{in}}}}}}}}\times 100 \%$$

$${{{{{{\rm{H}}}}}}}_{2}/{{{{{\rm{CO}}}}}}\,{{{{{\rm{ratio}}}}}}=\frac{{\left[{{{{{{\rm{H}}}}}}}_{2}\right]}_{{{{{{\rm{out}}}}}}}}{{\left[{{{{{\rm{CO}}}}}}\right]}_{{{{{{\rm{out}}}}}}}}\times 100 \%$$

$${{{{{\rm{Selectivity}}}}}}\,{{{{{{\rm{H}}}}}}}_{2}=\frac{{2\times \left[{{{{{{\rm{H}}}}}}}_{2}\right]}_{{{{{{\rm{out}}}}}}}}{{\left[{{{{{{\rm{CH}}}}}}}_{4}\right]}_{{{{{{\rm{in}}}}}}}-{\left[{{{{{{\rm{CH}}}}}}}_{4}\right]}_{{{{{{\rm{out}}}}}}}}\times 100 \%$$

$${{{{{\rm{Selectivity\; CO}}}}}}=\frac{{\left[{{{{{\rm{CO}}}}}}\right]}_{{{{{{\rm{out}}}}}}}}{{\left[{{{{{{\rm{CH}}}}}}}_{4}\right]}_{{{{{{\rm{in}}}}}}}-{\left[{{{{{{\rm{CH}}}}}}}_{4}\right]}_{{{{{{\rm{out}}}}}}}+{\left[{{{{{{\rm{CO}}}}}}}_{2}\right]}_{{{{{{\rm{in}}}}}}}-{\left[{{{{{{\rm{CO}}}}}}}_{2}\right]}_{{{{{{\rm{out}}}}}}}}\times 100 \%$$

where [CH_4_]_in_ and [CO_2_]_in_ denote the molar flow rates of the introduced CH_4_ and CO_2_, [CH_4_]_out_, [CO_2_]_out_, [H_2_]_out_, and [CO]_out_ denote the molar flow rates of CH_4_, CO_2_, H_2_, and CO in the tail gas.

### DFT computational method

Vienna Ab Initio Simulation Package (VASP) was adopted for our spin-polarized density functional theory calculations^55^. The ion-electron interaction and exchange-correlation were described using the projector-augmented plane-wave (PAW) approach and the Perdew–Burke–Ernzerhof (PBE) functional^56,57^. The plane-wave basis set with a cutoff energy of 400 eV was selected for the calculations. DFT+U correction (U_eff_ = 3.7 eV and 3.5 eV for Cr and Co, respectively) is considered to treat the 3*d* orbital electrons^58,59^.

A 2 × 2 supercell slab with eight atom layers is established for LaCrO_3_ 010 and 001 surfaces. Cr atoms are replaced with Ni or Co atoms depending on the specific configuration (Supplementary Figs. 18 and 19) and the topmost O atoms (O_α_) were removed to ensure the open of 3d orbitals of the catalytically active transition metal elements. The bottom two layers were fixed while all other atoms were relaxed until the maximum force was less than 0.02 eV Å^−1^. The vacuum space along the projection direction of the top surface was more than 15 Å to avoid the interactions between period images. The surface Brillouin zone was sampled by a 2 × 2 × 1 k points mesh.

## Supplementary information

Peer Review File

Supplementary Information

## Data Availability

The authors declare that all data supporting the findings of this study are available within the article and its supplementary information files, and from the corresponding author on request.
