# Supplementary material for: Highly active and thermostable submonolayer La(NiCo)OΔ catalyst stabilized by a perovskite LaCrO3 support
Source: Commun Chem. 2022 Jun 3;5:70. doi: 10.1038/s42004-022-00686-4 (PMC9814614; doi:10.1038/s42004-022-00686-4)
Supplement: Supplementary file 2 — Supplementary Information [file 42004_2022_686_MOESM2_ESM.pdf]

Supplementary Information

Highly active and thermostable submonolayer La(NiCo)O_Δ catalyst stabilized by a perovskite $LaCrO_3$ support

Tingting Zhao, Jiankang Zhao, Xuyingnan Tao, Haoran Yu, Ming Li, Jie Zeng, Haiqian Wang*

Hefei National Laboratory for Physical Science at the Microscale, University of Science and Technology of China, Hefei, Anhui 230026, People's Republic of China

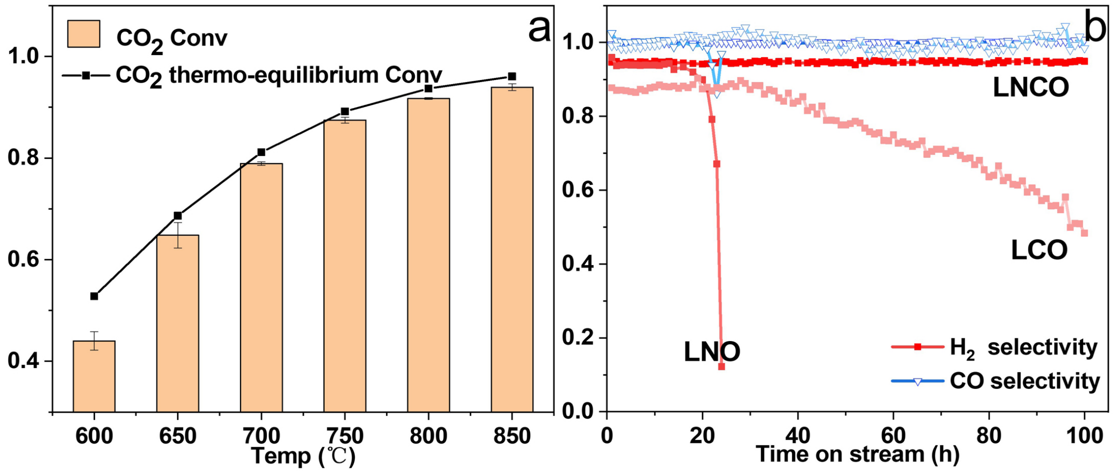

Supplementary Figure 1: DRM performance over LNCO, LNO, and LCO catalysts. a, temperature-dependent CO_2 conversion between 600 – 850 ^oC over LNCO catalyst. **b**, selectivities of H_2 and CO of LNCO, LNO, and LCO catalysts at 750 ^oC. Conditions: CH_4: CO_2 = 1: 1, total flow rate = 60 sccm (GHSV = 1.2×10^4 mL·g_{cat}^{-1}·h^{-1}). LNCO, LNO, and LCO represent $LaNi_{0.05}Co_{0.05}Cr_{0.9}O_3$, $LaNi_{0.1}Cr_{0.9}O_3$, and $LaCo_{0.1}Cr_{0.9}O_3$ catalysts, respectively.

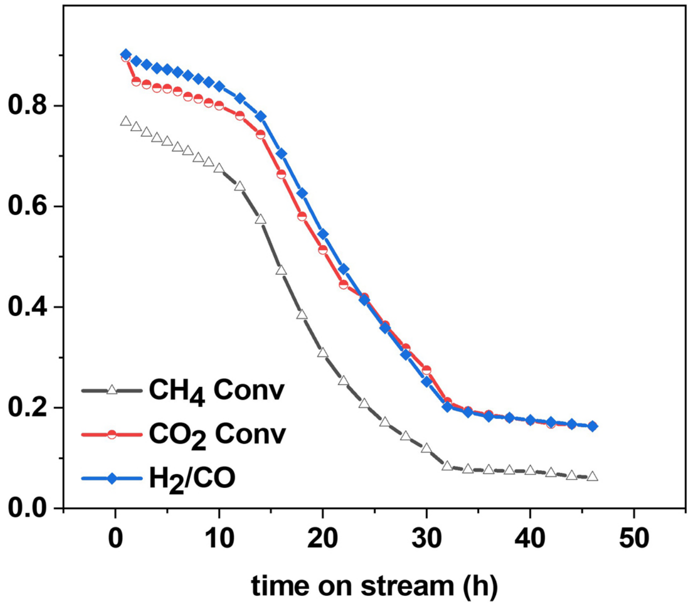

Supplementary Figure 2: DRM performance over impregnated NiCo@$LaCrO_3$ catalyst. Conditions: T = 750 ^oC, CH_4: CO_2 = 1: 1, total flow rate = 60 sccm (GHSV = 1.2×10^4 mL·g_{cat}^{-1}·h^{-1})

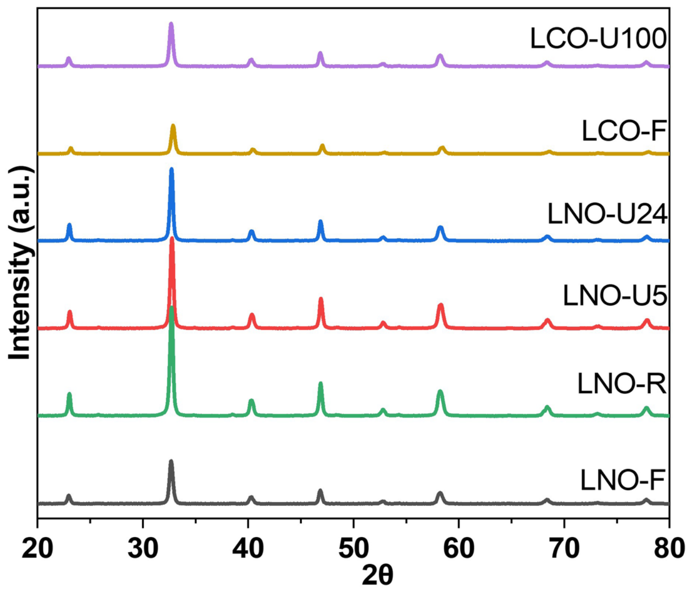

Supplementary Figure 3: XRD patterns of perovskite LNO and LCO samples. LNO-F, LNO-R, LNO-U5, and LNO-U24 represent fresh, H_2-reduced, used after 5 h time on stream, and used after 24 h time on stream $LaNi_{0.1}Cr_{0.9}O_3$ samples, respectively. LCO-F and LCO-U100 represent fresh and used after 100 h time on stream $LaCo_{0.1}Cr_{0.9}O_3$ samples, respectively.

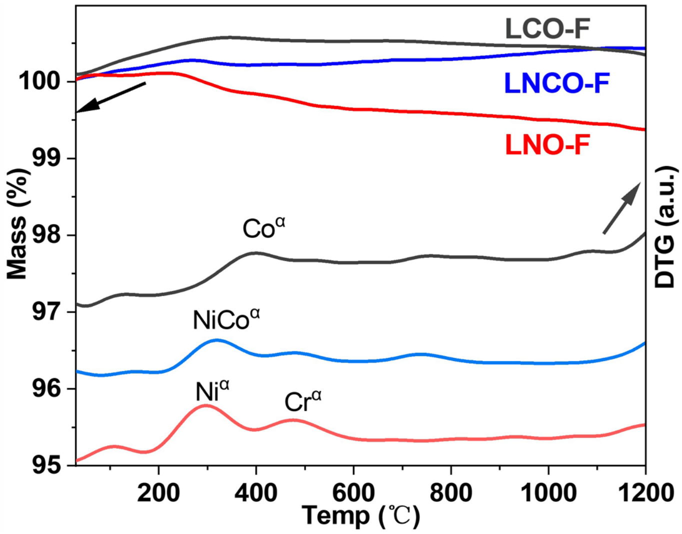

Supplementary Figure 4: TPR profiles of LNCO-F, LNO-F, and LCO-F. Ni$^{\alpha}$: Ni^{3+} to Ni^{2+}, Co$^{\alpha}$: Co^{3+} to Co^{2+}, NiCo$^{\alpha}$: Ni^{3+} and Co^{3+} to Ni^{2+} and Co^{2+}, Cr$^{\alpha}$: Cr^{6+} to Cr^{3+}. TPR measurement was carried out in 5 vol% H_2/N_2 atmosphere with a flow rate of 60 sccm. LNCO-F, LNO-F, and LCO-F represents fresh $LaNi_{0.05}Co_{0.05}Cr_{0.9}O_3$, $LaNi_{0.1}Cr_{0.9}O_3$, and $LaCo_{0.1}Cr_{0.9}O_3$ samples, respectively.

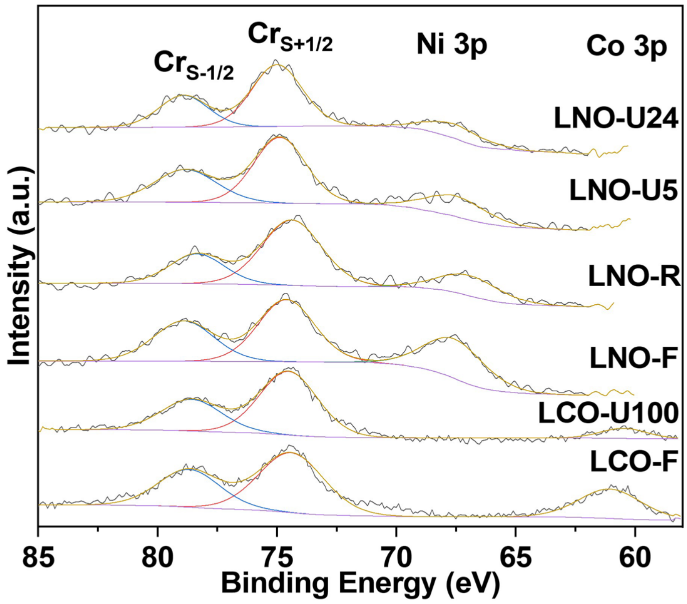

Supplementary Figure 5: Ni 3p, Co 3p, and Cr 3s XPS spectra of LNO and LCO samples. $Cr_{S+1/2}$ and $Cr_{S-1/2}$ denote intra-atomic multiplet splitting of Cr 3s with the remaining 3s electron coupled parallel and antiparallel to the 3d electrons, respectively. LNO-F, LNO-R, LNO-U5, and LNO-U24 represent fresh, H_2-reduced, used after 5 h time on stream, and used after 24 h time on stream $LaNi_{0.1}Cr_{0.9}O_3$ samples, respectively. LCO-F and LCO-U100 represent fresh and used after 100 h time on stream $LaCo_{0.1}Cr_{0.9}O_3$ samples, respectively.

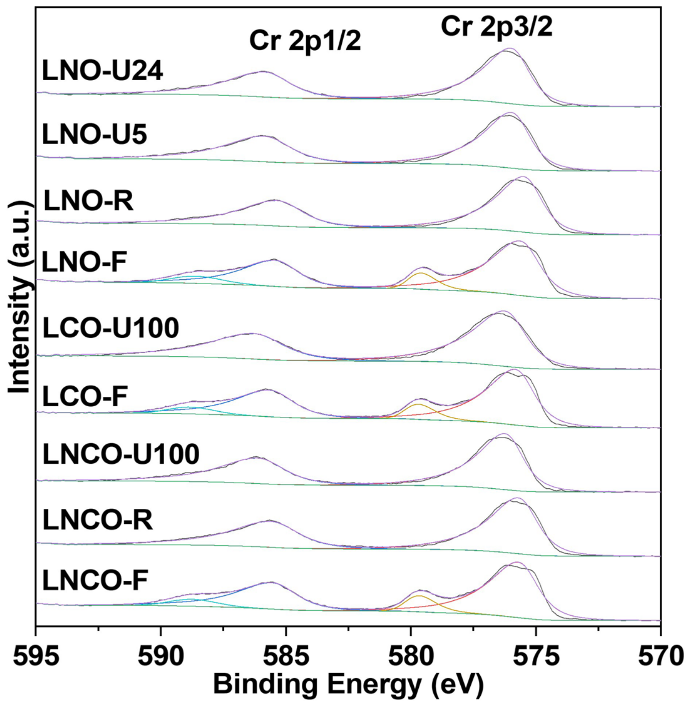

Supplementary Figure 6: Normalized Cr 2p spectra of LNC, LNO, and LCO samples of XPS. LNO-F, LNO-R, LNO-U5, and LNO-U24 represent fresh, H_2-reduced, used after 5 h time on stream, and used after 24 h time on stream $LaNi_{0.1}Cr_{0.9}O_3$ samples, respectively. LCO-F and LCO-U100 represent fresh and used after 100 h time on stream $LaCo_{0.1}Cr_{0.9}O_3$ samples, respectively. LNCO-F, LNCO-R, and LNCO-U100 represent fresh, H_2-reduced, and used after 100 h time on stream $LaNi_{0.05}Co_{0.05}Cr_{0.9}O_3$ samples, respectively.

The strong spin-orbit interaction splits the Cr 2p main peaks into Cr $2p_{3/2}$ and Cr $2p_{1/2}$ doublet separated by ~10 eV. The Cr 2p spectra of the fresh samples show two sets of Cr 2p doublets that belong to Cr^{3+} and Cr^{6+}, respectively. The peaks located at 575.7 and 585.6 eV can be ascribed to Cr^{3+}, while the peaks located at 579.8 and 588.9 eV arise from Cr^{6+} [1,2]. The relative concentration of Cr^{6+} accounts for about 14 at% of the total $Cr^{3+/6+}$ ions. No Cr(IV) compounds were detected by XRD should be because Cr^{6+} (possibly La_2CrO_6) mainly exists on the surface of the perovskite $LaNi_{0.05}Co_{0.05}Cr_{0.9}O_3$ and its absolute amount relative to the total mass is very small[3]. The Cr^{6+} peaks disappear once fresh samples undergo hydrogen reduction and DRM test, which consists with the XRD observation.

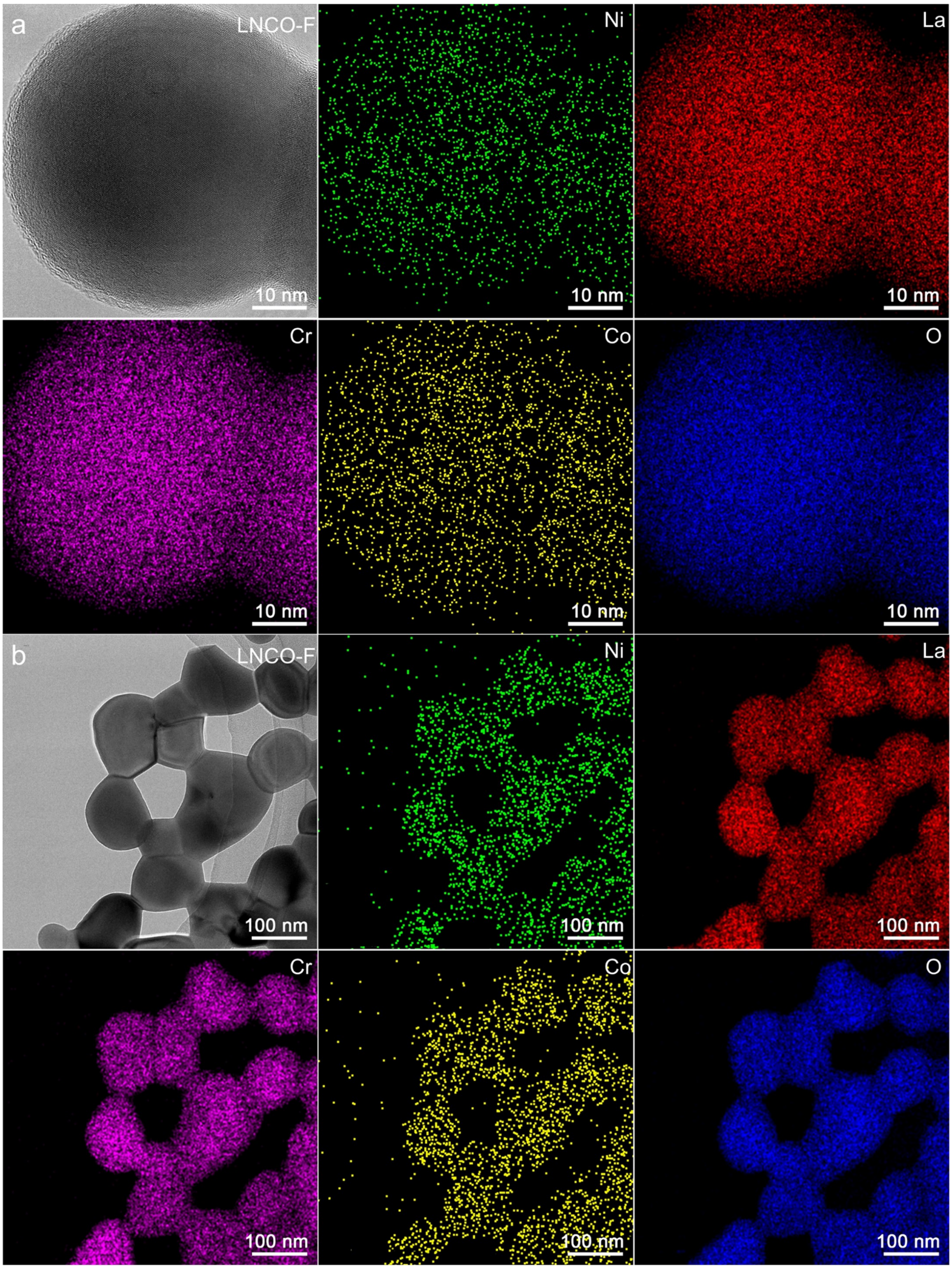

Supplementary Figure 7: TEM images and EDS-Mapping of LNCO-F. a, high magnification images to show the lattice structure and element distribution within a crystalline grain. **b**, low magnification images to show the element distribution in a larger area. LNCO-F represents fresh $LaNi_{0.05}Co_{0.05}Cr_{0.9}O_3$ sample.

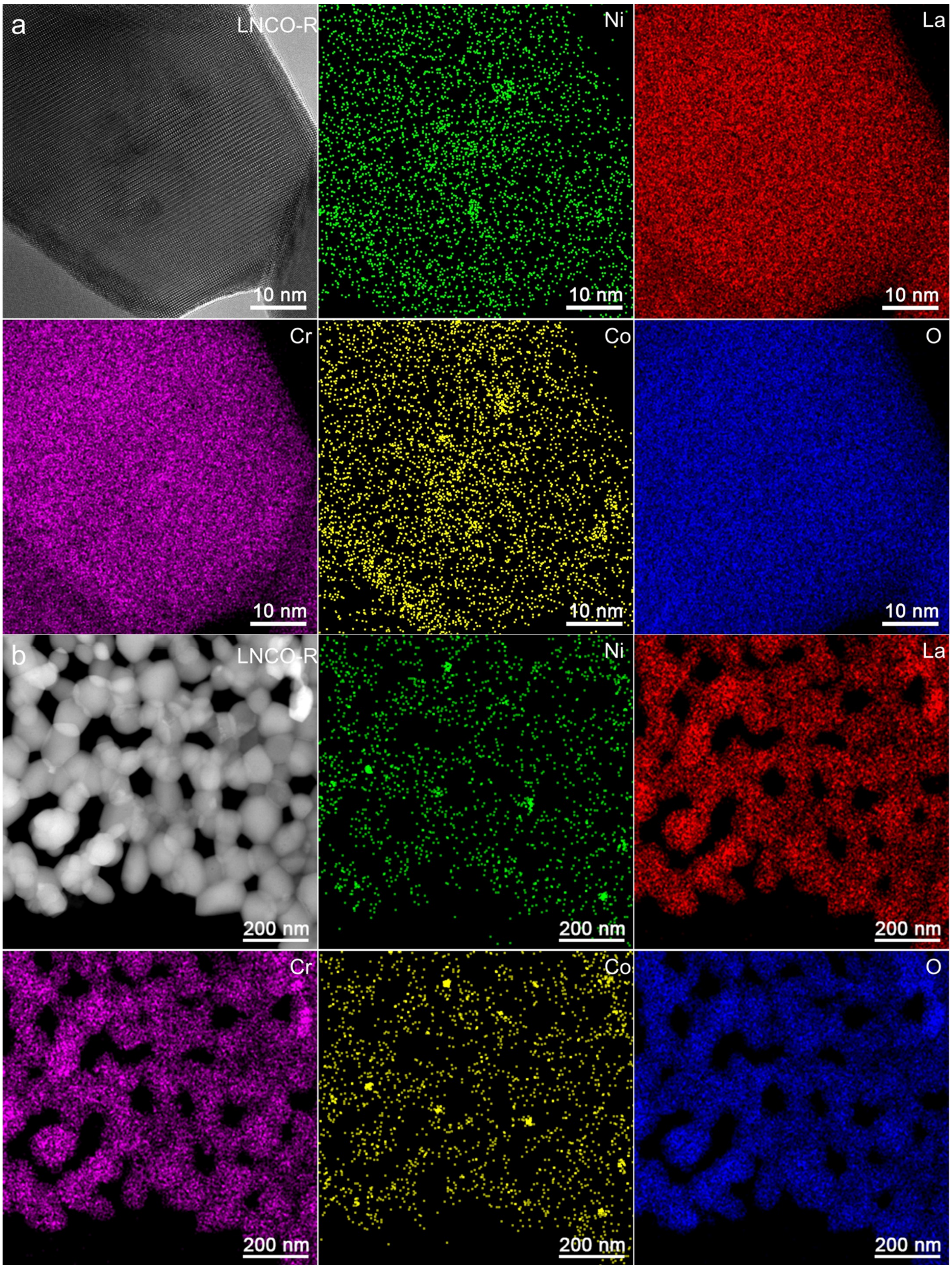

Supplementary Figure 8: TEM images and EDS-Mapping of LNCO-R. a, high magnification images to show the lattice structure and element distribution within a crystalline grain. **b**, low magnification images to show the element distribution and NiCo nanoparticles in a larger area. LNCO-R represents H_2-reduced $LaNi_{0.05}Co_{0.05}Cr_{0.9}O_3$ sample.

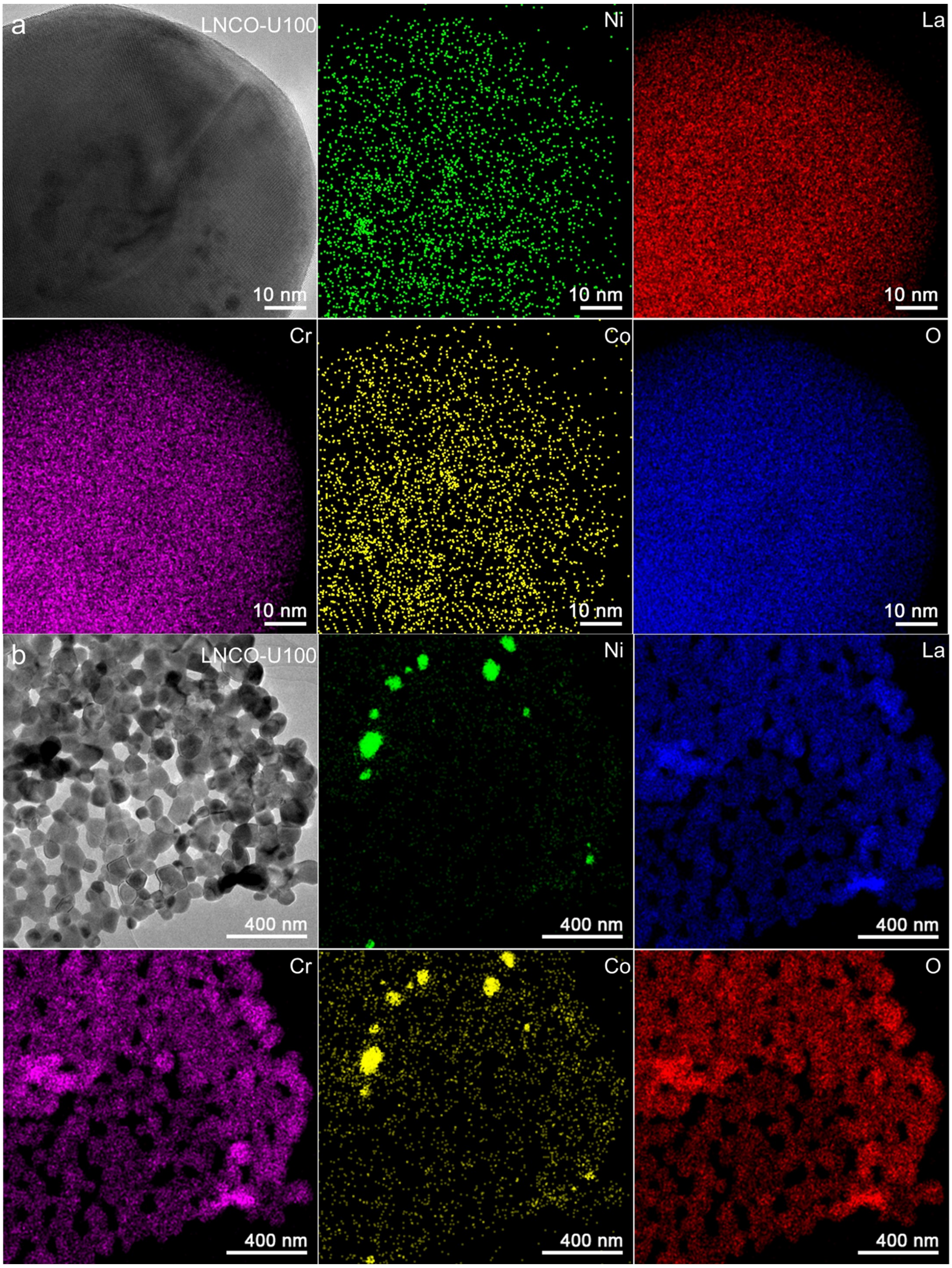

Supplementary Figure 9: TEM images and EDS-Mapping of LNCO-U100. **a**, high magnification images to show the lattice structure and element distribution within a crystalline grain. **b**, low magnification images to show the element distribution and NiCo nanoparticles in a larger area. LNCO-U100 represents used $LaNi_{0.05}Co_{0.05}Cr_{0.9}O_3$ sample after 100 h time on stream.

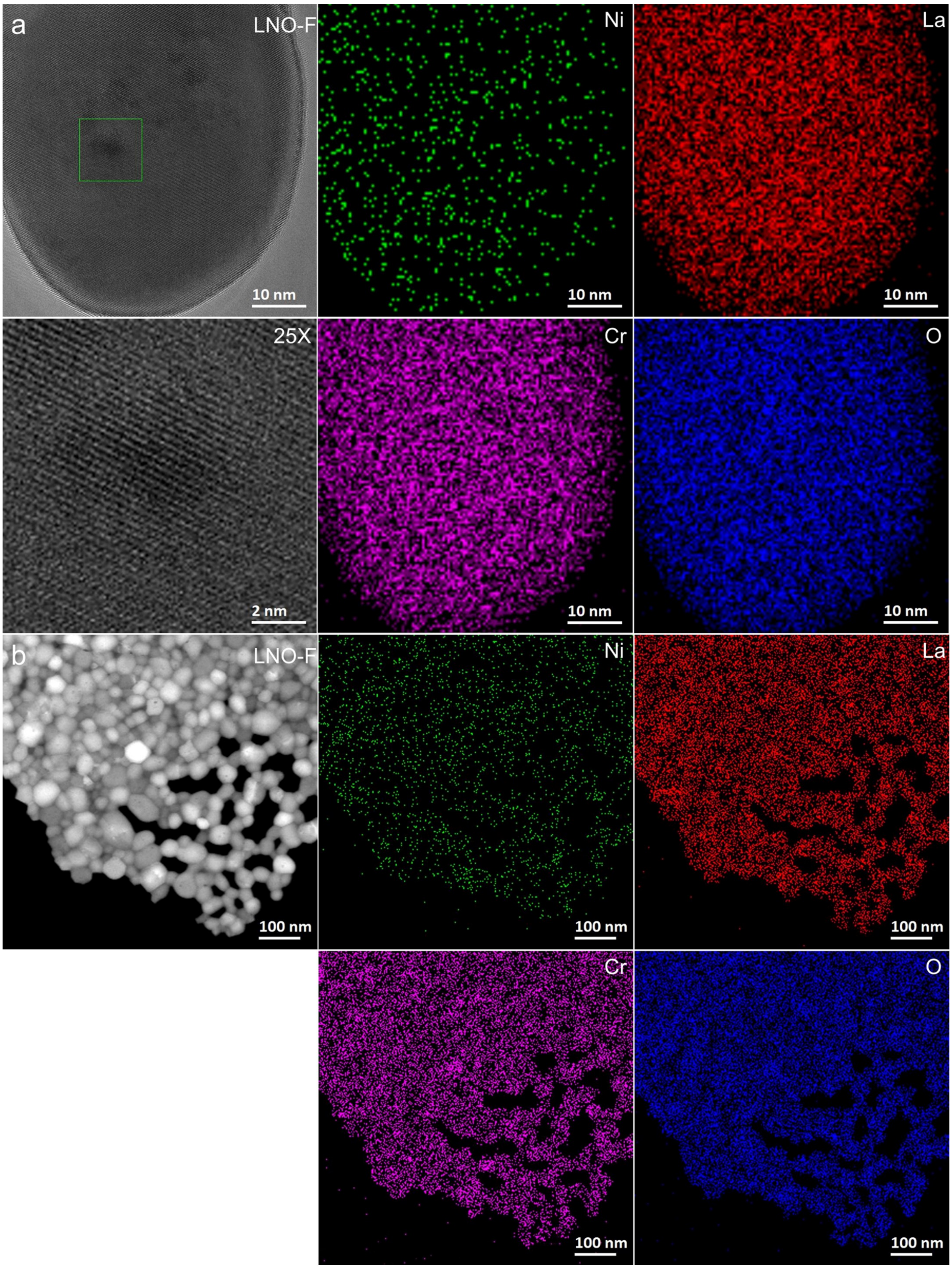

Supplementary Figure 10: TEM images and EDS-Mapping of LNO-F. a, high magnification images to show the lattice structure and element distribution within a crystalline grain. **b**, low magnification images to show the element distribution in a larger area. LNO-F represents fresh $LaNi_{0.1}Cr_{0.9}O_3$ sample.

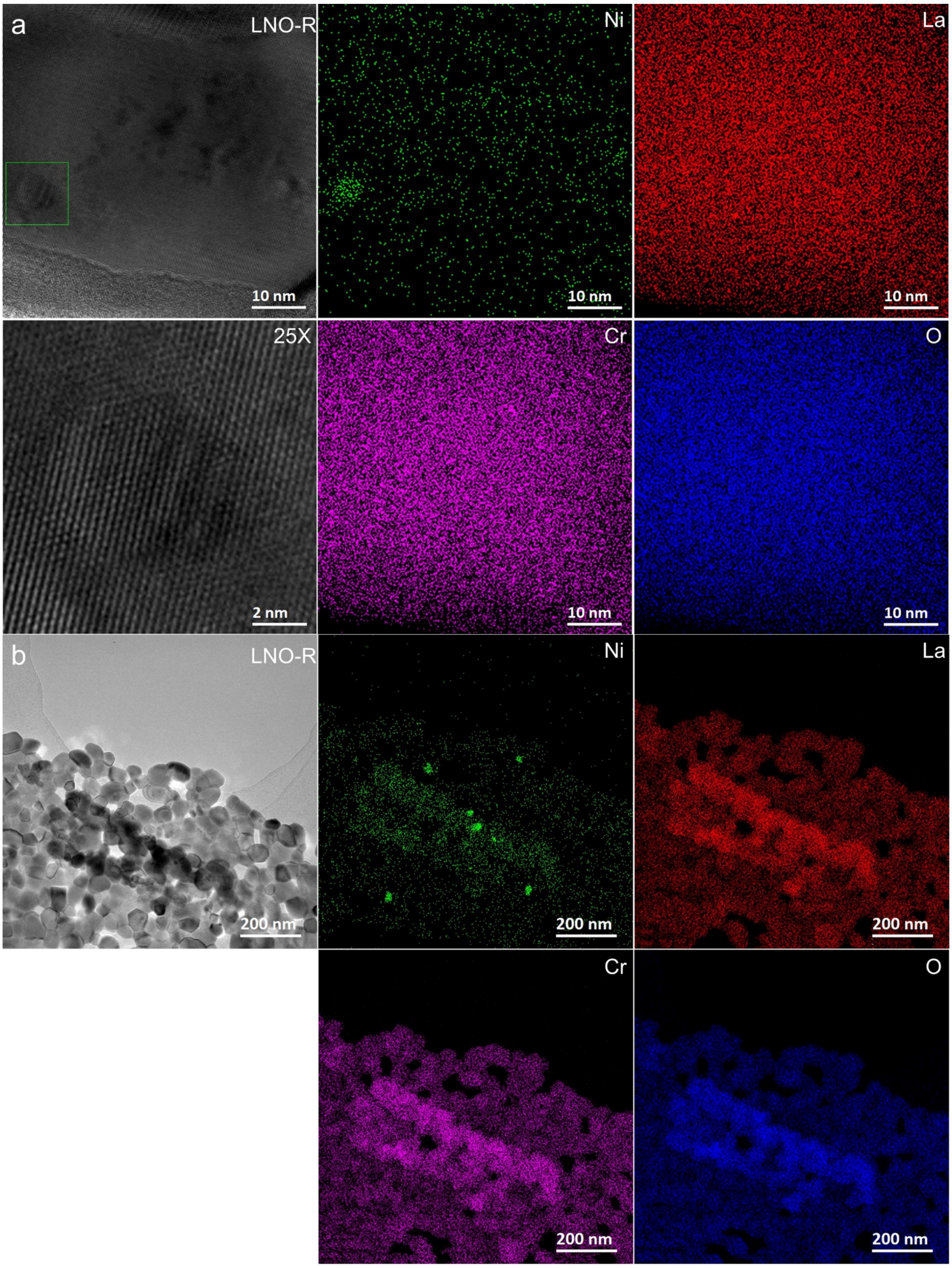

Supplementary Figure 11: TEM images and EDS-Mapping of LNO-R. a, high magnification images to show the lattice structure and element distribution within a crystalline grain. **b**, low magnification images to show the element distribution in a larger area. LNO-R represents H_2-reduced $LaNi_{0.1}Cr_{0.9}O_3$ sample.

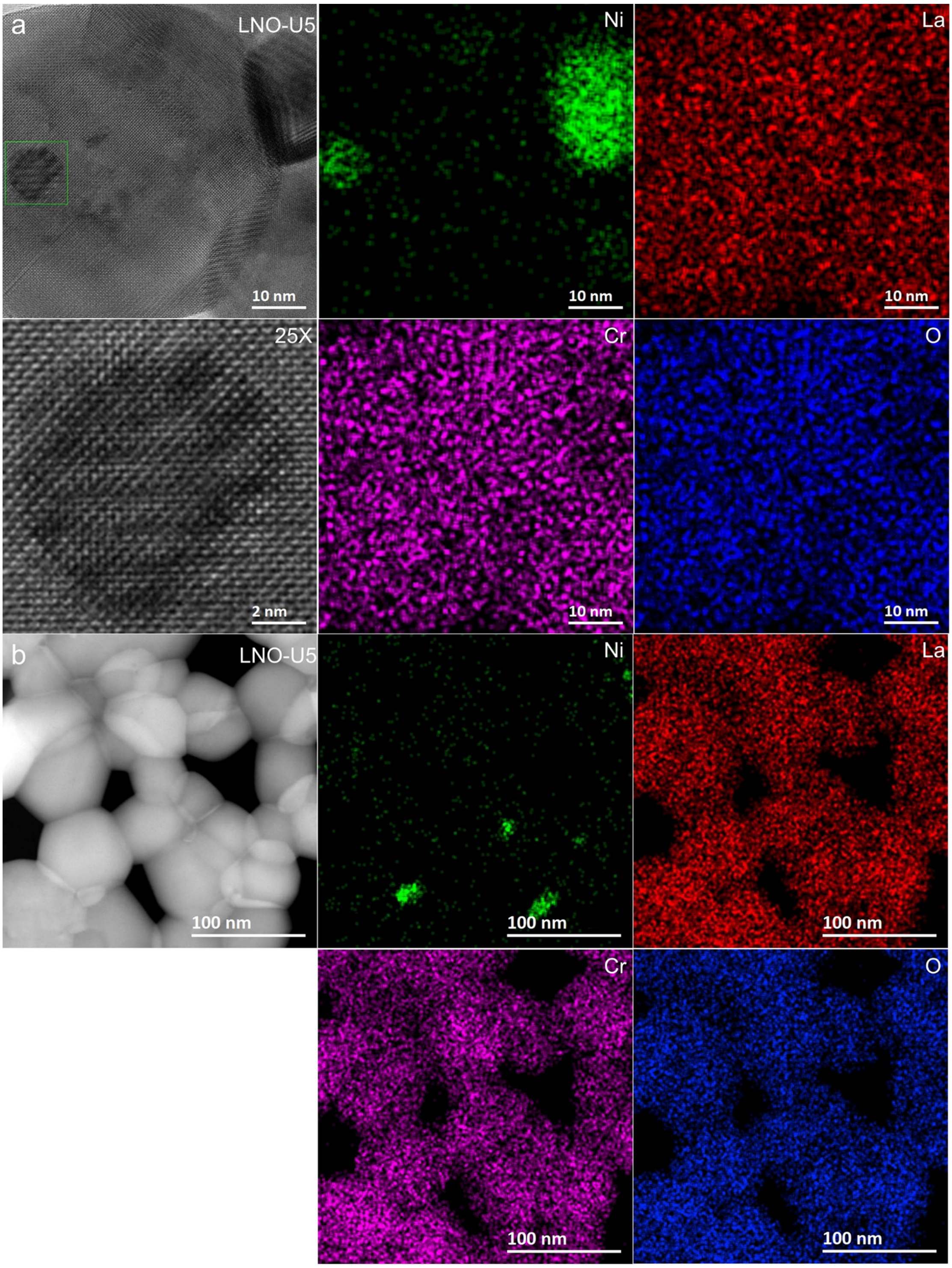

Supplementary Figure 12: TEM images and EDS-Mapping of LNO-U5. a, high magnification images to show the lattice structure and element distribution within a crystalline grain. **b**, low magnification images to show the element distribution in a larger area. LNO-U5 represents used $LaNi_{0.1}Cr_{0.9}O_3$ sample after 5 h time on stream.

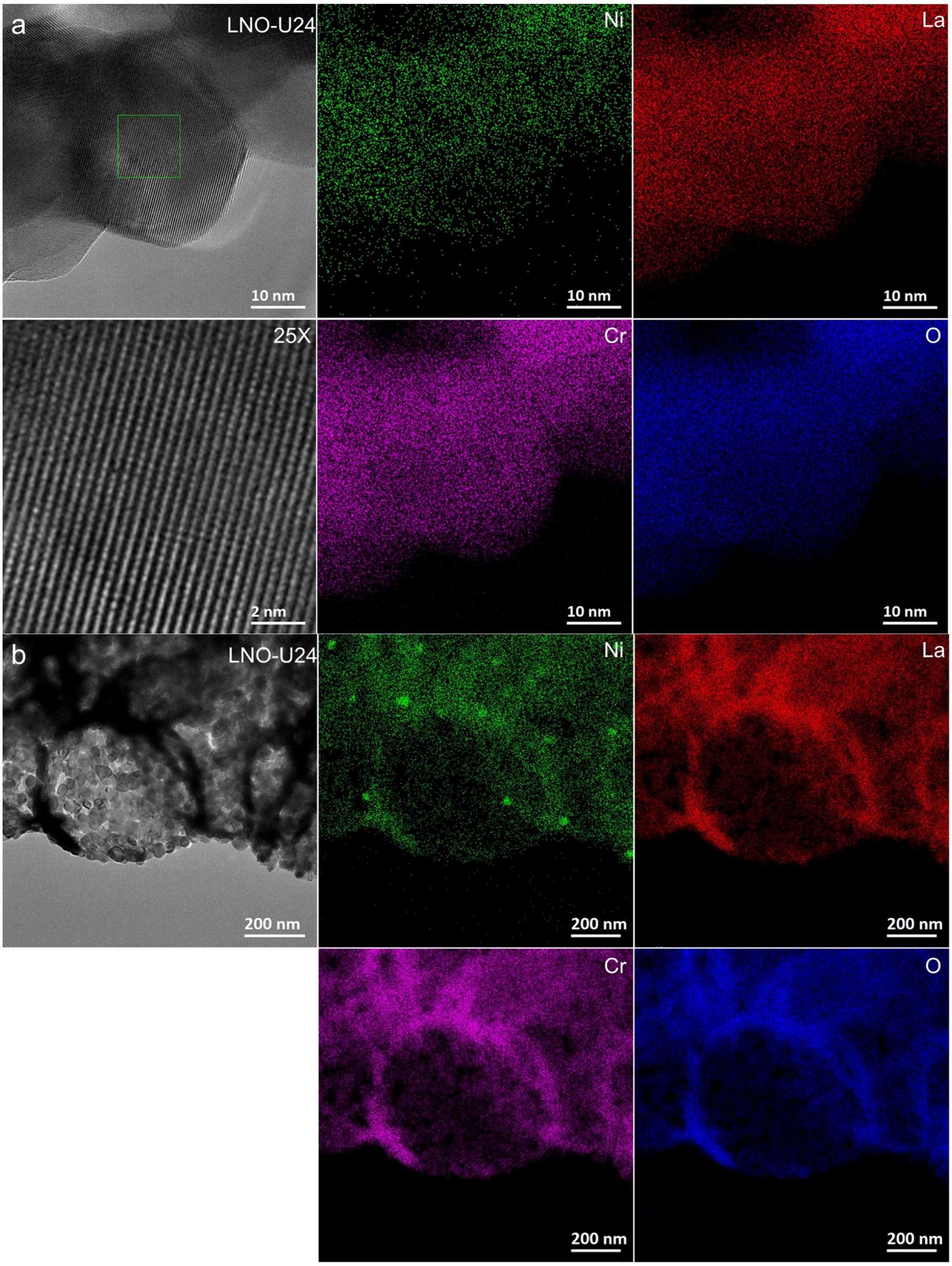

Supplementary Figure 13: TEM images and EDS-Mapping of LNO-U24. a, high magnification images to show the lattice structure and element distribution within a crystalline grain. **b**, low magnification images to show the element distribution in a larger area. LNO-U24 represents used $LaNi_{0.1}Cr_{0.9}O_3$ sample after 24 h time on stream.

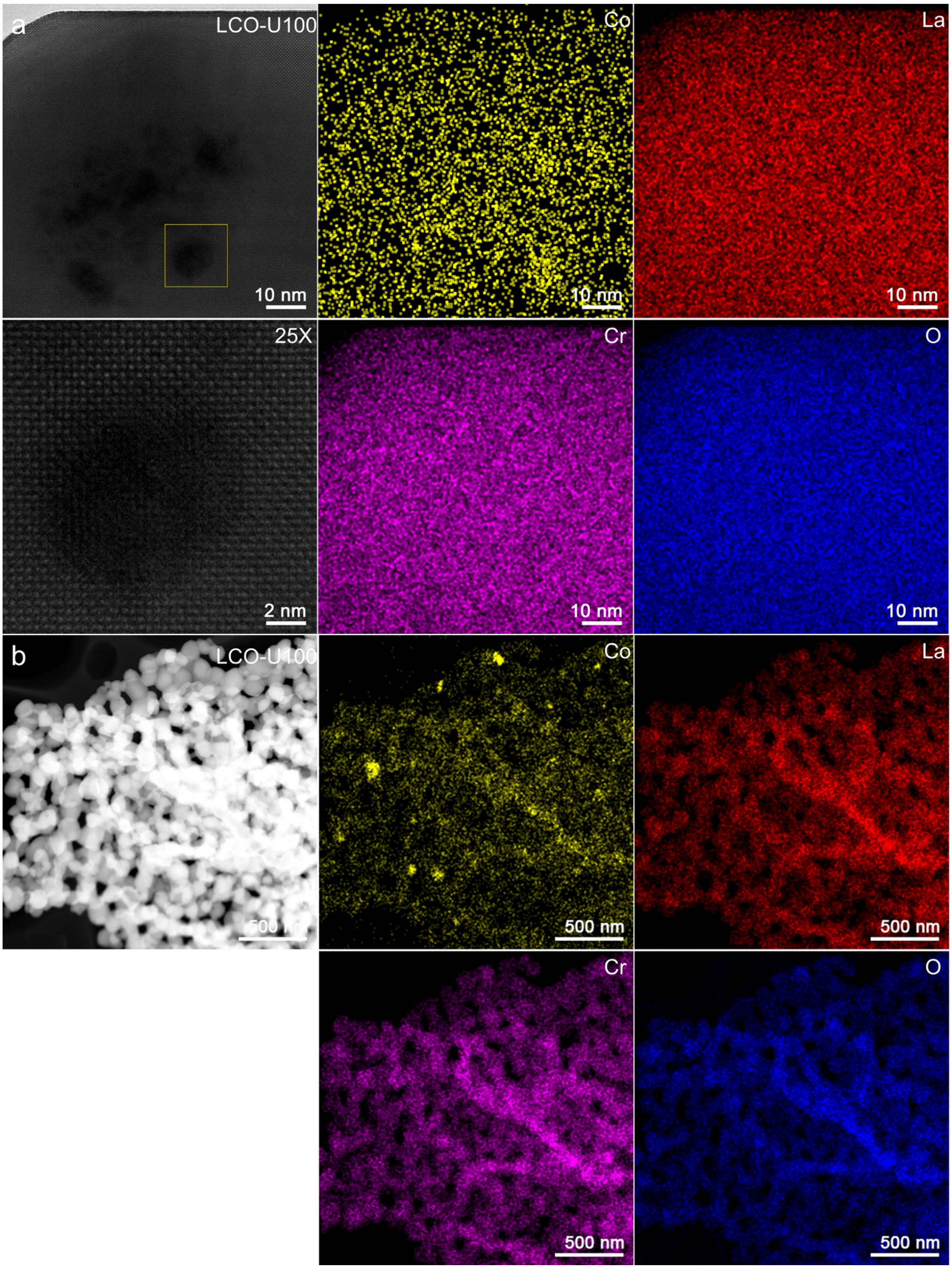

Supplementary Figure 14. TEM images and EDS-Mapping of LCO-U100. a, high magnification images to show the lattice structure and element distribution within a crystalline grain. **b**, low magnification images to show the element distribution in a larger area. LCO-U100 represents used $LaCo_{0.1}Cr_{0.9}O_3$ sample after 100 h time on stream.

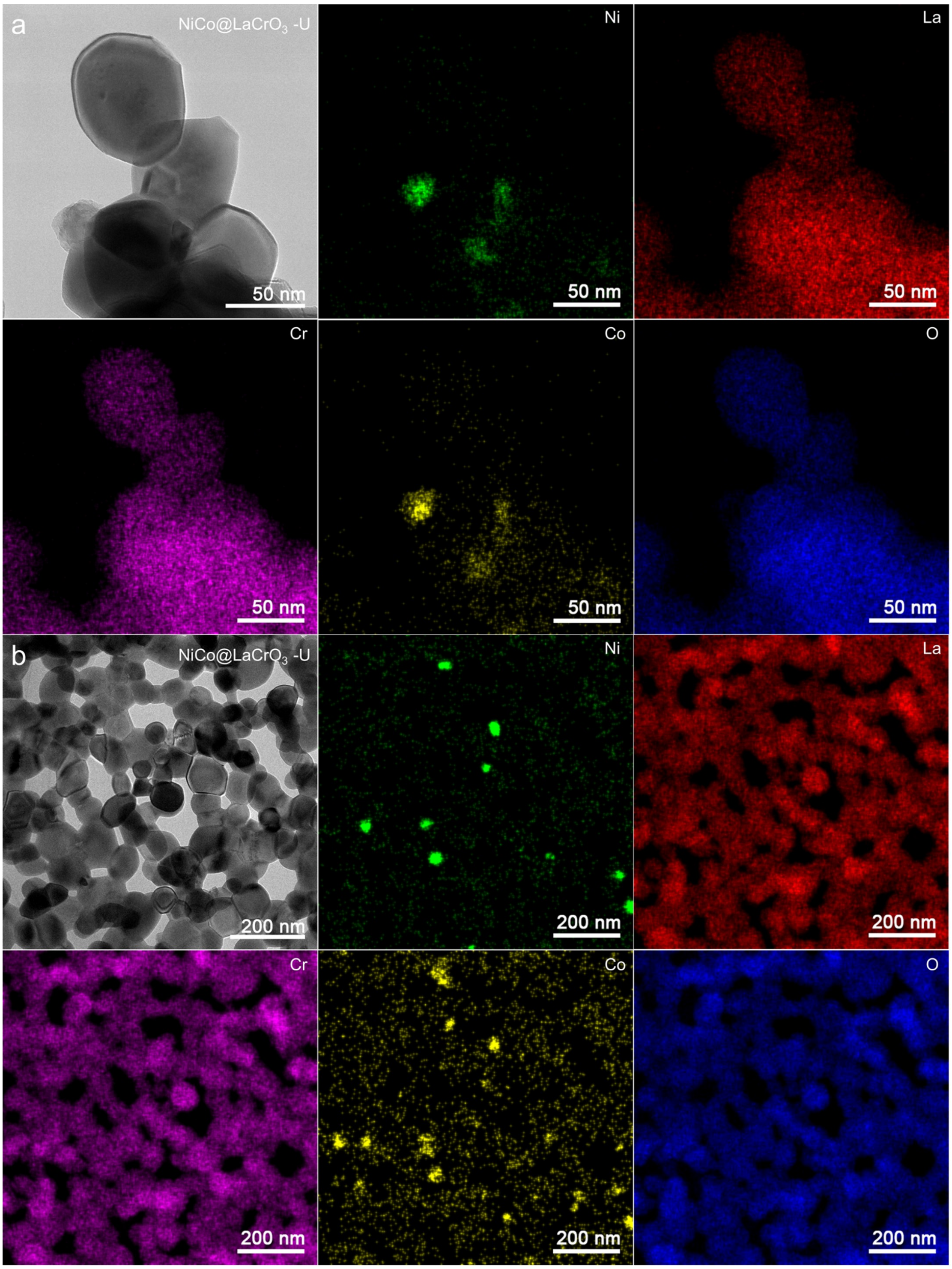

Supplementary Figure 15. TEM images and EDS-Mapping of NiCo@$LaCrO_3$ after 10 h time on stream, donated as NiCo@ $LaCrO_3$-U. a, high magnification images. **b**, low magnification images.

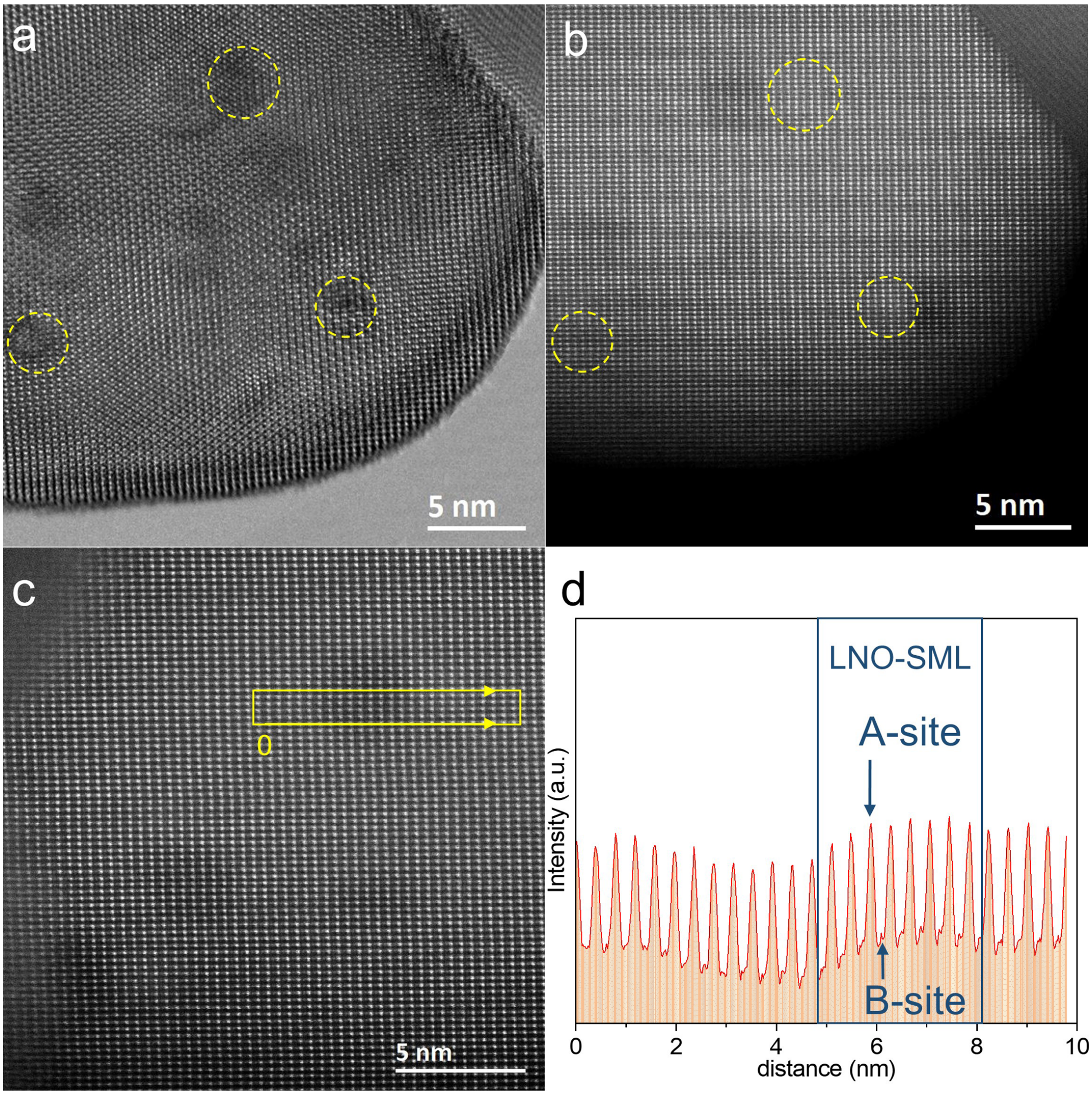

Supplementary Figure 16: (a) HR-TEM, (b) and (c) HAADF images of LNO-R sample. d, line profiles across A- and B-site elements as indicated in **c**. The brighter regions in **b** and **c** indicate LNO-SML regions. The surface direction is [001]. LNO-R represents H_2-reduced $LaNi_{0.1}Cr_{0.9}O_3$ sample. LNO-SML represents $LaNiO_{\Delta}$ submonolayer.

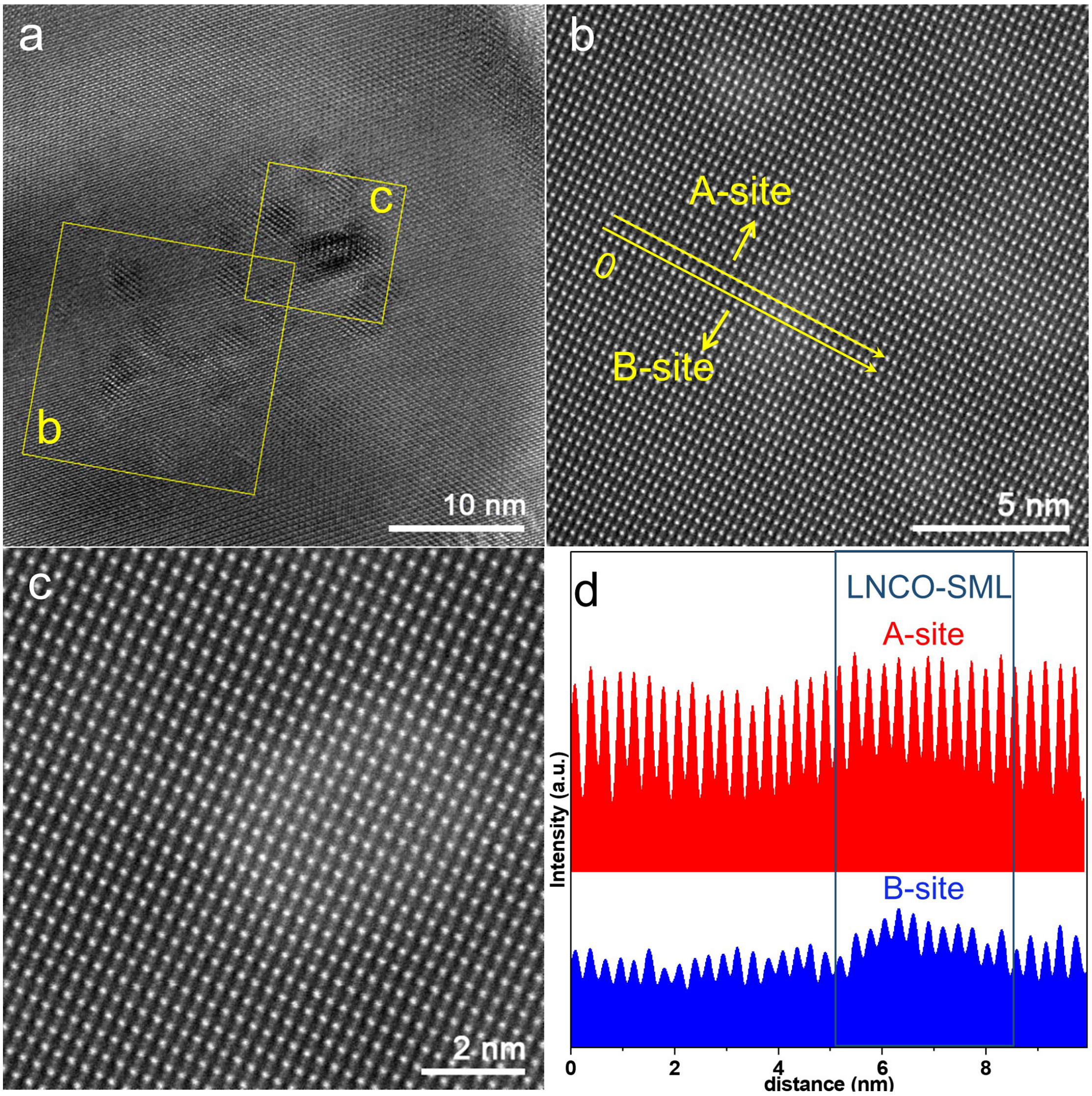

Supplementary Figure 17: (a) HR-TEM, (b) and (c) HAADF images of LNCO-U100 sample. d, line profiles across A- and B-site elements as indicated in **b**. The brighter regions in **b** and **c** indicate LNCO-SML regions. The surface direction is [020]. LNCO-U100 represents used $LaNi_{0.05}Co_{0.05}Cr_{0.9}O_3$ sample after 100 h time on stream. LNCO-SML represents $La(NiCo)O_\Delta$ submonolayer.

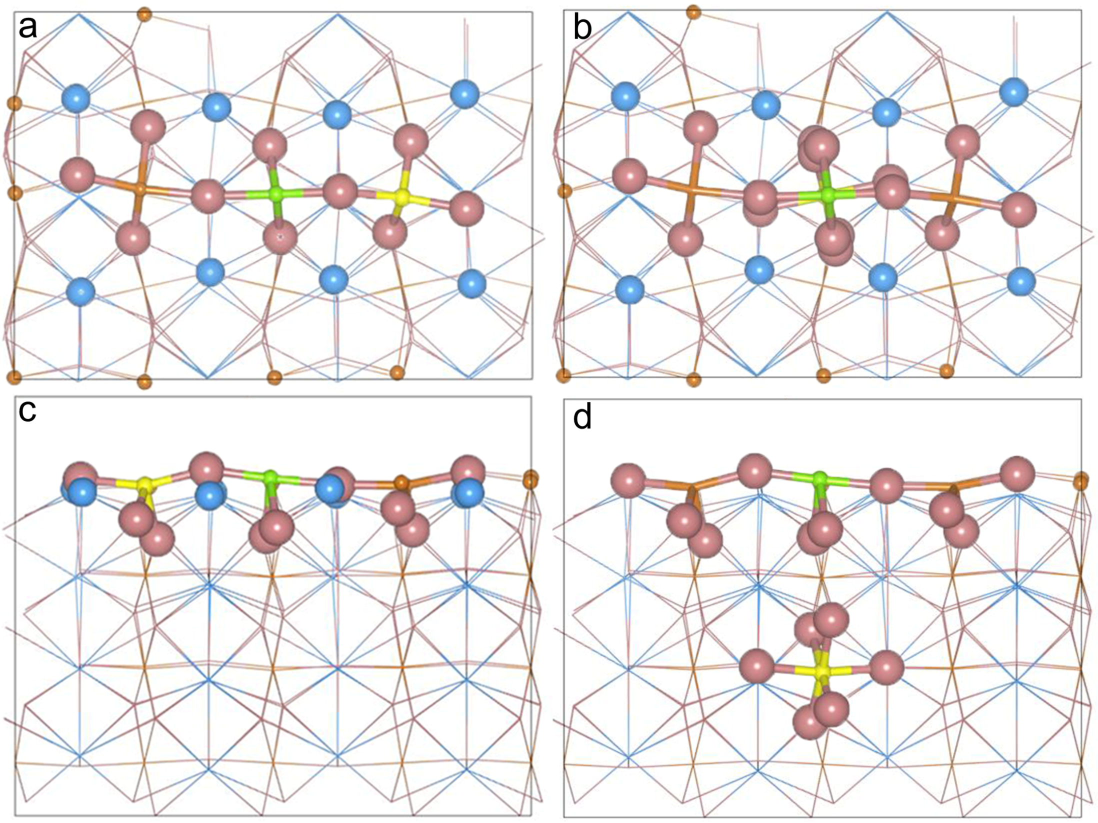

Supplementary Figure 18: Top (a) and front (c) views of the configuration with B-site neighboring Ni and Co atoms on the $LaCrO_3$ top layer (010 surface); top (b) and front (d) views of the configuration one Ni atom on the top surface and one Co atom in the bulk of $LaCrO_3$. Red, blue, orange, green and yellow balls represent oxygen, lanthanum, chromium, nickel and cobalt atoms, respectively.

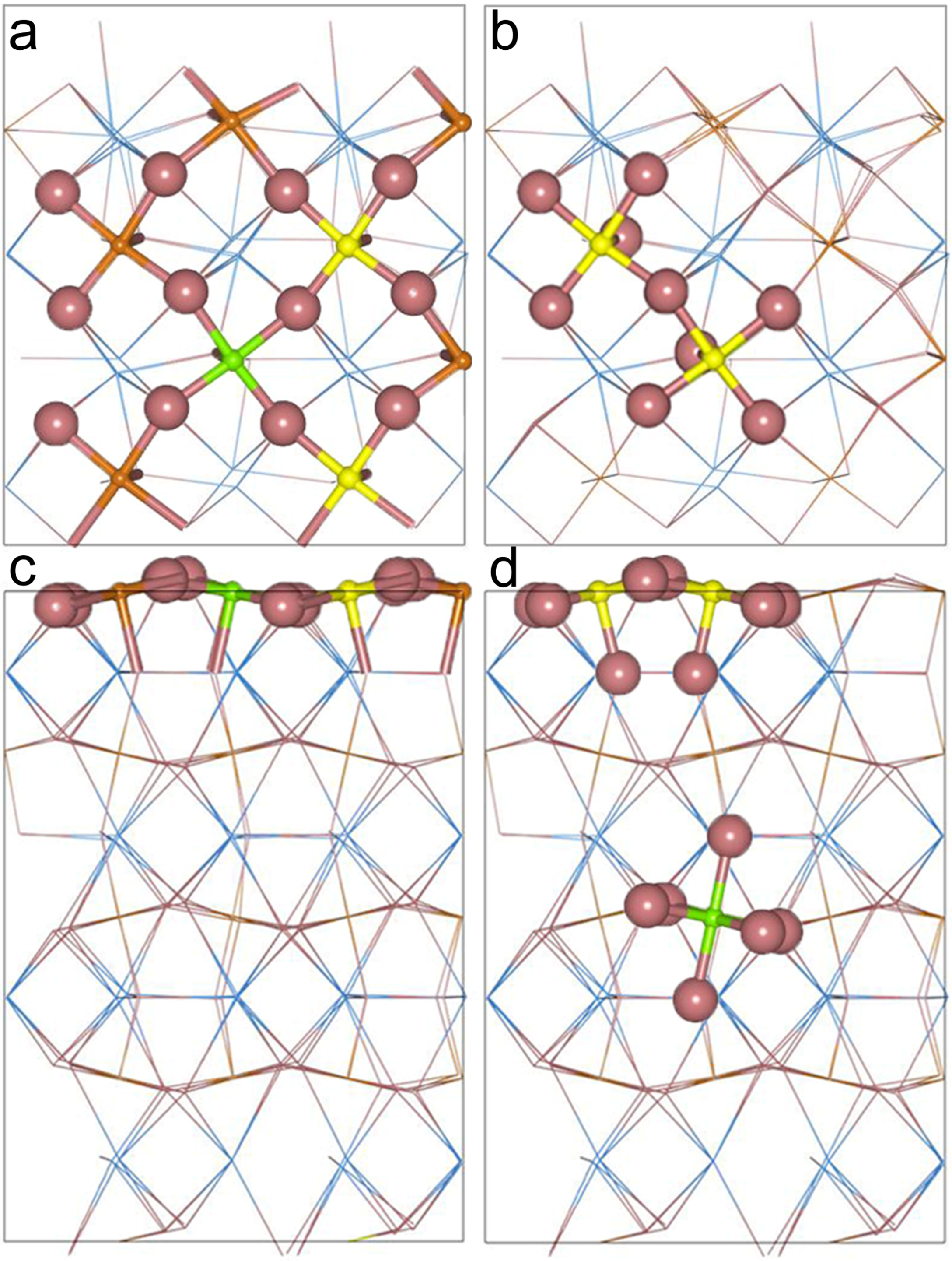

Supplementary Figure 19: Top (a) and front (c) views of the configuration with B-site neighboring Co, Ni, Co atoms on the $LaCrO_3$ top layer (001 surface); top (b) and front (d) views of the configuration with two Co atoms on the top surface and one Ni atom in the bulk of $LaCrO_3$. Red, orange, green and yellow balls represent oxygen, chromium, nickel and cobalt atoms, respectively.

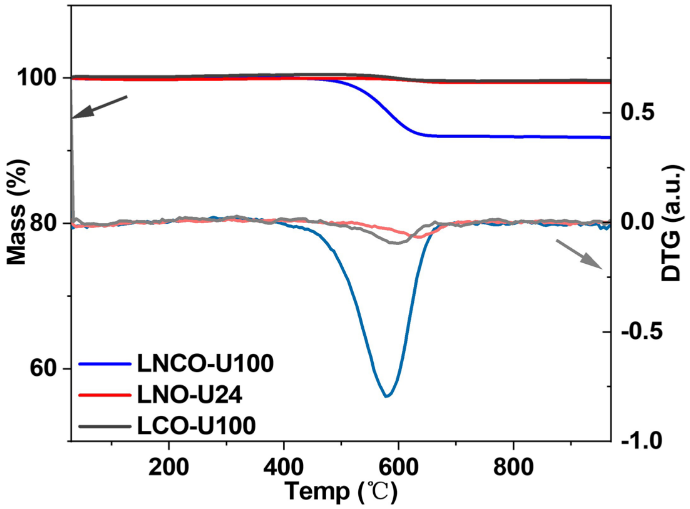

Supplementary Figure 20: TPO profiles of LNCO-U100, LNO-U24, and LCO-U100. TPO measurement was carried out in dry air with a flow rate of 60 sccm. LNCO-U100, LNO-U24, and LCO-U100 represent used $LaNi_{0.05}Co_{0.05}Cr_{0.9}O_3$ sample after 100 h time on stream, used $LaNi_{0.1}Cr_{0.9}O_3$ sample after 24 h time on stream, and used $LaCo_{0.1}Cr_{0.9}O_3$ sample after 100 h time on stream, respectively

Supplementary Table 1: ΔE of Cr 3s multiplet splitting, and the atomic ratio of Ni/ Cr and Co/Cr determined by Ni 3p, Co 3p and Cr 3s XPS spectra in Fig. 3.

sample ID	ΔE	Ni/Cr (atomic ratio)	Co/Cr (atomic ratio)
$LaNi_{0.05}Co_{0.05}Cr_{0.9}O_3$			
LNCO-F	4.2	0.11	0.048
LNCO-R	4.0	0.076	0.014
LNCO-U100	3.9	0.070	0.010
$LaNi_{0.1}Cr_{0.9}O_3$			
LNO-F	4.3	0.11	--
LNO-R	4.0	0.060	--
LNO-U5	4.0	0.061	--
LNO-U24	3.9	0.047	--
$LaCo_{0.1}Cr_{0.9}O_3$			
LCO-F	4.3	--	0.10
LCO-U100	4.1	--	0.033

DFT computational method

Vienna Ab Initio Simulation Package (VASP) was adopted for our spin-polarized density functional theory calculations[4]. The ion-electron interaction and exchange-correlation were described using the projector-augmented plane wave (PAW) approach and the Perdew-Burke-Ernzerhof (PBE) functional[5,6]. The plane-wave basis set with a cutoff energy of 400 eV was selected for the calculations. DFT+U correction (U_{eff} = 3.7 eV and 3.5 eV for Cr and Co, respectively) is considered to treat the 3*d* orbital electrons[7,8].

A 2×2 supercell slab with eight atom layers is established for $LaCrO_3$ 010 and 001 surfaces. Cr atoms are replaced with Ni or Co atoms depending on the specific configuration (Supplementary Figure 18-19) and the topmost O atoms (O_α) were removed to ensure the open of 3d orbitals of the catalytically active transition metal elements. The bottom two layers were fixed while all other atoms were relaxed until the maximum force was less than 0.02 eV $Å^{-1}$. The vacuum space along the projection direction of the top surface was more than 15 Å to avoid the interactions between period images. The surface Brillouin zone was sampled by a 2×2×1 k points mesh.

Supplementary References

1 Álvarez-Galván, M. C. *et al.* Methyl ethyl ketone combustion over La-transition metal (Cr, Co, Ni, Mn) perovskites. *Applied Catalysis B: Environmental* **92**, 445-453, doi:https://doi.org/10.1016/j.apcatb.2009.09.006 (2009).

2 Liu, X. *et al.* Mixed valence state and electrical conductivity of La1−xSrxCrO3. *Journal of Alloys and Compounds* **305**, 21-23, doi:https://doi.org/10.1016/S0925-8388(00)00735-0 (2000).

3 Rida, K. *et al.* Effect of calcination temperature on the structural characteristics and catalytic activity for propene combustion of sol–gel derived lanthanum chromite perovskite. *Applied Catalysis A: General* **327**, 173-179, doi:https://doi.org/10.1016/j.apcata.2007.05.015 (2007).

4 Kresse, G. & Furthmüller, J. Efficiency of ab-initio total energy calculations for metals and semiconductors using a plane-wave basis set. *Computational Materials Science* **6**, 15-50, doi:https://doi.org/10.1016/0927-0256(96)00008-0 (1996).

5 Kresse, G. & Joubert, D. From ultrasoft pseudopotentials to the projector augmented-wave method. *Physical Review B* **59**, 1758-1775, doi:10.1103/PhysRevB.59.1758 (1999).

6 Perdew, J. P., Burke, K. & Ernzerhof, M. Generalized Gradient Approximation Made Simple. *Physical Review Letters* **77**, 3865-3868, doi:10.1103/PhysRevLett.77.3865 (1996).

7 Dabaghmanesh, S., Sarmadian, N., Neyts, E. C. & Partoens, B. A first principles study of p-type defects in LaCrO3. *Physical Chemistry Chemical Physics* **19**, 22870-22876, doi:10.1039/C7CP03575F (2017).

8 García-Mota, M. *et al.* Importance of Correlation in Determining Electrocatalytic Oxygen Evolution Activity on Cobalt Oxides. *The Journal of Physical Chemistry C* **116**, 21077-21082, doi:10.1021/jp306303y (2012).
